# Phase Transitions and Water Splitting Applications of 2D Transition Metal Dichalcogenides and Metal Phosphorous Trichalcogenides

**DOI:** 10.1002/advs.202002284

**Published:** 2021-03-11

**Authors:** Tingke Rao, Huide Wang, Yu‐Jia Zeng, Zhinan Guo, Han Zhang, Wugang Liao

**Affiliations:** ^1^ College of Electronic and Information Engineering Institute of Microscale Optoelectronics Shenzhen University Shenzhen 518060 P. R. China; ^2^ Institute of Microscale Optoelectronics College of Physics and Optoelectronic Engineering Shenzhen University Shenzhen 518060 P. R. China

**Keywords:** 2D phase transition materials, electrochemical catalyst, metal phosphorous trichalcogenide, photocatalyst, transition metal dichalcogenide

## Abstract

2D layered materials turn out to be the most attractive hotspot in materials for their unique physical and chemical properties. A special class of 2D layered material refers to materials exhibiting phase transition based on environment variables. Among these materials, transition metal dichalcogenides (TMDs) act as a promising alternative for their unique combination of atomic‐scale thickness, direct bandgap, significant spin–orbit coupling and prominent electronic and mechanical properties, enabling them to be applied for fundamental studies as catalyst materials. Metal phosphorous trichalcogenides (MPTs), as another potential catalytic 2D phase transition material, have been employed for their unusual intercalation behavior and electrochemical properties, which act as a secondary electrode in lithium batteries. The preparation of 2D TMD and MPT materials has been extensively conducted by engineering their intrinsic structures at the atomic scale. In this study, advanced synthesis methods of preparing 2D TMD and MPT materials are tested, and their properties are investigated, with stress placed on their phase transition. The surge of this type of report is associated with water‐splitting catalysis and other catalytic purposes. This study aims to be a guideline to explore the mentioned 2D TMD and MPT materials for their catalytic applications.

## Graphical abstract

2D phase transition materials, including transition metal dichalcogenides (TMDs) and metal phosphorous trichalcogenides (MPTs), attract researchers' interests for their unique combination of large surface area, direct bandgap, intrigue chemical, and mechanical properties. Great efforts are devoted to preparing 2D TMD and MPT materials by engineering their intrinsic structures at the atomic scale, aiming to achieve active catalyst toward catalytic purposes.

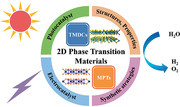

## 1 Introduction

Environmental pollution and energy shortage are posing increasing threats to the earth. To solve the energy crisis, green fuel (e.g., H_2_ synthesized from water) is an ideal solution. Though it has been a long time since the electrochemical reaction was proposed, the overall energy efficiency remains questionable for practical application with a slow reaction rate. Besides, large‐scale production is still challenging to achieve since excellent catalysts exhibiting large surface area, chemical stability, proper activation energy, and catalytic efficiency are lacked. Precious metals (e.g., Platinum) showed outstanding catalytic performance with numerous electrochemical catalytic purposes.^[^ ^1^ ^]^ However, their widespread application is mostly restricted by the scarcity and prohibited price. Accordingly, the development of alternative electrocatalysts composed of low‐cost and adequate properties is urgently required.

One of the potential catalysts reported is 2D nanomaterial, i.e., a class of material exhibiting single or few layers of atoms yet no dangling bond on the surface. The mentioned materials comprise graphene,^[^ ^2^, ^3^, ^4^, ^5^, ^6^, ^7^, ^8^ ^]^ transition metal dichalcogenides (TMDs),^[^ ^9^, ^10^, ^11^, ^12^, ^13^, ^14^, ^15^, ^16^, ^17^, ^18^ ^]^ Xenes (e.g., phosphorene,^[^ ^19^, ^20^, ^21^, ^22^, ^23^, ^24^, ^25^ ^]^ bismuthene,^[^ ^26^, ^27^, ^28^, ^29^, ^30^ ^]^ antimonene,^[^ ^31^, ^32^, ^33^, ^34^, ^35^, ^36^ ^]^ tellurene,^[^ ^37^, ^38^, ^39^ ^]^ and borophene^[^ ^40^, ^41^, ^42^ ^]^), transition metal carbide/transition metal nitride (Mxene),^[^ ^43^, ^44^, ^45^ ^]^ graphyne,^[^ ^46^, ^47^, ^48^, ^49^, ^50^ ^]^ perovskites,^[^ ^51^, ^52^ ^]^ metal phosphorous trichalcogenides (MPTs),^[^ ^53^, ^54^ ^]^ etc. The mentioned materials have aroused huge attention for their unique mechanical, electronic, and catalytic properties, especially in (opto)electronics,^[^ ^55^, ^56^, ^57^ ^]^ sensing,^[^ ^58^, ^59^ ^]^ energy,^[^ ^60^, ^61^, ^62^ ^]^ and biomedicine.^[^ ^63^, ^64^ ^]^ Much of the highlighted and intrigue properties attracting interests are their anisotropy in electrical, optical, and mechanical behaviors. To be specific, those exhibiting phase transition possessing intrigue properties including tunable bandgaps and dipole ordering, show great potential to be exfoliated into 2D catalysts with the tunable electrical and optical properties. The tunable bandgap, conductivity and stability lead to the increase in the efficiency of electrochemical and photo‐induced applications.

Among the 2D phase transition materials, TMDs aroused the most attention for their structure and strong covalent bond, composed of a metal plane enclosed by two anionic chalcogen planes enlarged into infinite layers, bonded each other by van der Waals (vdW) force. For instance, single‐layered MoS_2_ is identified in two distinct symmetries, i.e., the 2H (trigonal prismatic D_3h_) and 1T (octahedral O_h_) phases depending on the arrangement of its S atoms. The two phases should exhibit completely different electronic properties, with the 2H phase as the semiconducting and the 1T phase metallic. The two phases are capable of converting one to the other via intralayer atomic plane gliding, which involves a transversal displacement of one of the S planes. Furthermore, the bandgap of 2H‐MoS_2_ can be enlarged from 1.29 to 1.9 eV from multilayer to monolayer.^[^ ^65^ ^]^

**Figure 1:**
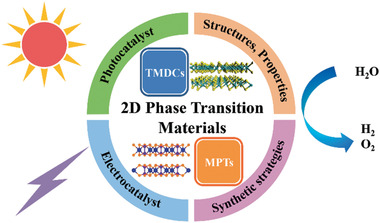
Schematic illustration of 2D phase transition materials as catalysts.

Layered MPTs, dominated by divalent metal cations that are stabilized in octahedral sulfur or selenium framework, have different lattices (monoclinic or rhombohedral) depending on stacking order. Strong ionic bonds between [P_2_Ch_6_]^4−^ and metallic cations lead to the generations of more functional groups on the surface, thereby helping achieve catalytic purposes. Moreover, synergistic P into the chalcogen structure broadened the bandgap to a broader range (1.3–3.5 eV ^[^ ^66^ ^]^), compared with the bandgap range of TMD layered materials,^[^ ^67^ ^]^ so the optical and electrical properties can be conveniently regulated. Active site [P_2_Ch_6_]^4−^ on the surface was reported to improve the catalytic activity. Moreover, the magnetism and electric polarization arising from spontaneous spin and dipole ordering fall to four categories, i.e., ferromagnetic (FM) by parallel spin, antiferromagnetic (AFM) by antiparallel spin, ferroelectric (FE) by parallel dipole and antiferroelectric (AFE) by antiparallel.^[^ ^68^ ^]^ Properties corresponded with ordering structures can be easily tuned by strain, interface interaction, external fields, etc. The tuned 2D phase transition materials are employed in catalytic circumstances (**Figure** **1**).

Theory study of 2D phase transition materials underpins the process from structure to application. For instance, the actual dynamical process of phase transition involving intralayer atomic plane gliding has been experimentally proven recently. Suppose one is to consider the possibility of intentionally introducing the phase transition in single‐layered materials in a controllable manner. In that case, this phase transition atomic process and its boundary structures should be corroborated to develop future low‐dimensional devices reliably.

In this study, the 2D phase transition materials are discussed from atomic and electronic structure, preparation methods to catalytic applications. Given the layer pattern, the structure of 2D TMD is categorized into 1T, 1T’, 2H, and 3R. Meanwhile, the system of MPT is divided into AFM, FM, FE, and AFE. Up to now, obtaining monolayered, uncontaminated and surface‐active 2D phase transition material remains a big challenge, which directs our way to summarize the growth of atomic layered 2D phase transition materials. Considering the mentioned intrigue physical and chemical properties of layered materials, we also elaborate their catalytic applications. The insights on the performance focus on numerous aspects, such as the influence of the metal cation, chalcogenide, metal alloying, and exfoliation. This study is intended as a comprehensive baseline for the anticipated new wave of researchers who aim to explore the mentioned 2D layered materials and their advancement in electrochemistry and energy applications.

## 2 Structure and Electrical Properties of Phase Transition 2D Materials

### 2.1 Structure of 2D TMDs and MPTs

On the whole, different phases of 2D materials correspond to a range of crystal structures. Thus, the following section introduces the crystallographic structure characteristics of 2D phase transition TMDs and MPTs, respectively.

#### 2.1.1 TMDs

**Figure 2:**
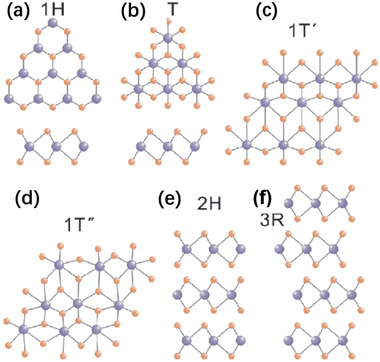
Structures of 2D TMDs. a) 1H, b) 1T, c) 1T’, d) 1T’’, e) 2H, and f) 3R phases. Reproduced with permission.^[^ ^69^ ^]^ Copyright 2019, Science in China Press.

The molecular formula of the TMDs family is MX_2_ (where M represents the transition metal and X represents S, Se, or Te). Monolayer MX_2_ is composed in the form of X‐M‐X, where the M atomic layer is sandwiched between two X atomic layers, and X and M atoms are covalently connected. Bulk and multilayered 2D MX_2_ are constructed by monolayer units that vertically stack via vdWs force. Also, the weak interlayer vdWs force connection contributes to different structures of TMDs. To be specific, monolayer MX_2_ generally involves two basic phases, i.e., the trigonal prismatic phase (1H phase if monolayer or 2H phase if multilayer) and the octahedral phase (1T phase), as given in **Figure** **2**a,b. The sequence of 1H phase MX_2_ is AbA (A represents S, Se, or Te, and b represents the transition metal), while AbC (A, C represents S, Se, or Te, and b represents the transition metal) for 1T phase MX_2_. The 1T phase MX_2_ can be considered a result of horizontal shift from the initial position of one of the sulfur atomic layers in the 1H phase MX_2_. Besides, the 1T’ phase and 1T’’ phase MX_2_ can be obtained by the distortion of 1T phase MX_2_, as shown in Figure 2c,d. 2H phase and 3R phase MX_2_ can be obtained by stacking MX_2_ of single layer 1H phase in a different order, which is manifested as the stacked sequences of ABA and ABC, respectively (Figure 2e,f).

The phase transition of TMDs is equivalent to the rearrangement of the electrons in the transition metal atoms in TMDs. Take the phase transition of 1H‐MoS_2_ to 1T‐MoS_2_ as an example. The d‐orbitals of the Mo atoms in 1H‐MoS_2_ are divided into three energy levels, i.e., two orbitals of *d*_*xy*,*yz*_, two orbitals of $d_{x^{2}-y^{2},xy}$, and one orbital of $d_{z^{2}}$.^[^ ^70^ ^]^ Alternatively, the d‐orbitals of the Mo atoms in 1T‐MoS_2_ are divided into two energy levels, i.e., two orbitals of $d_{x^{2}-y^{2},z^{2}}$, and three orbitals of *d*_*xy*,*yz*, *zx*_. The two electrons in outer d‐orbitals of Mo atom are filled into the same $d_{z^{2}}$ orbital for 1H‐MoS_2_. Consequently, 1H‐MoS_2_ exhibits semiconductor properties as a result of empty d‐orbitals. Meanwhile, for 1T‐MoS_2_, the mentioned two electrons are filled into any two orbitals of the three *d*_*xy*,*yz*, *zx*_ orbitals, respectively. High mobility of the single electron in the d‐orbitals endows 1T‐MoS_2_ metallic properties. That is, the phase transition of TMDs can be achieved by changing the concentration of electrons in the TMDs. Furthermore, the total energy of the two electrons in the outer d‐orbitals of Mo atoms in the 1H‐MoS_2_ is lower than that of the 1T‐MoS_2_, thus a more stable 1H/2H phase is formed compared with the 1T phase.

When tailored to 2D scale, TMD materials exhibit intrigue surface properties derived from the monolayered unit. As demonstrated from the numerical calculation, the basal plane of pure H‐MoS_2_ is inert and thus hinders the catalytic application.^[^ ^71^ ^]^ Zang et al. revealed the basal plane of H‐MoS_2_ invert by carbon‐induced modulation. The $4d_{z^{2}}$ orbitals of Mo atoms and the 3*p*_*x*,*y*_ orbitals of S atoms exhibit the unmatched charge interaction with water molecule originated from the steric effect ($4d_{z^{2}}$) and unbefitting orbital orientation (3*p*_*x*,*y*_) in the basal plane.^[^ ^71^ ^]^ Alternatively, Jaramillo et al. demonstrated TMD possess active sites at the edge.^[^ ^72^ ^]^ Numerical calculation results have demonstrated active sites of other H‐phase TMDs exist on edge of the basal plane, highlighting the significance of basal plane activation.^[^ ^73^ ^]^

#### 2.1.2 MPTs

MPTs, as one kind of layered phase transition materials, show wide application prospect in energy storage, fuel conversion, catalysts, etc. The metal cations in layered MPTs compounds are mainly divalent (+2) and can be replaced by both monovalent and trivalent metal cations, inconsistent with the TMDs metal cations, which exhibit a tetravalent (+4) oxidation state. There are two main forms of MPTs: M^II^PX_3_ (M^II^ includes Mg^2+^, Mn^2+^, Fe^2+^, Co^2+^, Ni^2+^, Zn^2+^, Pd^2+^, Cd^2+^, Sn^2+^, Hg^2+^, etc.; X includes S and Se) and M^I^M^III^P_2_X_6_ (M^I^ includes Ag^+^ and Cu^+^; M^III^ includes Sc^3+^, V^3+^, Cr^3+^, Al^3+^, Ga^3+^, In^3+^, Bi^3+^, etc.). It is indicated that MPTs may have more types of compounds than TMDs. From the crystal structure, MPT materials have the following common characteristics: i) Metal cations (M^II^ or M^I^M^III^) and [P_2_S_6_]^4−^ or [P_2_Se_6_]^4−^ anions bind to each other by strong ionic bonds. ii) The [P_2_S_6_]^4−^ or [P_2_Se_6_]^4−^ anion layer constitutes the skeleton of a monolayer MPTs, while the metal cations are distributed around [P_2_S_6_]^4−^ or [P_2_Se_6_]^4−^ in a honeycomb arrangement. Accordingly, MPTs are commonly expressed as M_2_P_2_S_6_ or M_2_P_2_Se_6_.

##### M^II^PX_3_ Compounds

**Figure 3:**
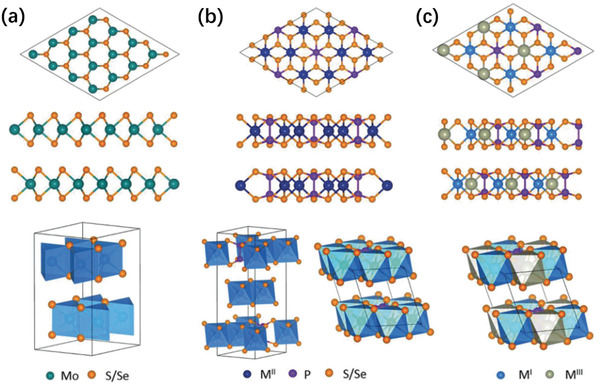
The typical crystal structures of a) MoS_2_, b) M^II^PX_3_, and c) M^I^M^III^P_2_X_6_, including the top view (top), side view (middle), and crystal phase (down). Reproduced with permission.^[^ ^74^ ^]^ Copyright 2018, Wiley‐VCH.

M^II^PTs can be considered one‐third of the M sites in the transition metal disulfide (MS_2_) was replaced by the p–p pairs (P_2_), then its composition becomes M_2/3_ (P_2_)_1/3_S_2_, i.e., M_2_P_2_S_6_. Here, each P atom is tetrahedral with three S atoms, while each S atom coordinates with two M^II^ sites and is covalently bound to one P atom. The typical layered structures of MoS_2_ and M^II^PTs are shown in **Figure** **3**a,b, respectively. However, significant differences are identified between M^II^PS_3_ and M^II^PSe_3_ in symmetry and crystal structure. The atomic layers of M^II^PS_3_ are in the C2/m space group, presenting the sequence of layer accumulation of “AAA”. However, due to the increase of the distance of P—Se bond and the angle of Se—P—Se bond, the atomic layers of M^II^PSe_3_ are reported to be largely located in the R‐3 space group, mainly showing the sequence of layer accumulation of “ABC”. Alternatively, Hg_2_P_2_Se_6_ displays an unusual structure, and its P–P pairs tilt, causing the octahedral cage between [P_2_Se_6_]^4−^ units to be distorted, so its stacking sequence is “ABAB”.

##### M^I^M^III^P_2_X_6_ Compounds

The M^II^ metal cations in the M^II^PTs compounds can be replaced by both M^I^ and M^III^ metal cations to form M^I^M^III^P_2_X_6_. The homo‐charge substitution can easily occur if the size of metal ions is nearly the same. After the substitution, the crystal structure is consistent with that before the substitution, and M^I^ and M^III^ usually appear alternately at the sites of original M^II^. But CuAlP_2_Se_6_, AgAlP_2_Se_6_, and AgCrP_2_Se_6_ compounds exhibit a random cationic order. Most M^I^M^III^P_2_S_6_ exhibit “ABAB” stacking sequences with different spatial groups, and their structure can be easily regulated. Under the large size difference between M^I^ and M^III^ metal cation, M^I^M^III^P_2_S_6_ has distorted crystal structure, such as AgVP_2_S_6_ and AgCrP_2_S_6_ compounds.^[^ ^74^ ^]^ The typical layered structures of M^I^M^III^P_2_X_6_ are illustrated in Figure 3c.

### 2.2 Electronic Properties of 2D TMDs

The property of TMD is intrigued among 2D materials for its unique electronic properties. All 2H and 1T phased TMDs in group V are indicated to be metallic with states at the Fermi level. A same metallic property was reported for Group IV TMDs with fewer states near the Fermi level and larger bandgaps.^[^ ^75^ ^]^ Group VI TMDs was found to be semiconducting in their thermodynamically stable 2H phase, while those 1T‐phased were metallic. While group X TMDs are also semiconducting in the 1T structure with a bandgap. All the mentioned results on the single‐layered basal planes confirm what is known about their bulk metallicity from existing experimental studies.

Absence of crystallographic symmetry, together with 2D quantum confinement and strong spin–orbit coupling, lead to many unique properties in layered TMDs, including the direct bandgap,^[^ ^76^ ^]^ optical harmonic generation,^[^ ^77^ ^]^ spin‐valley coupling,^[^ ^78^ ^]^ magnetoelectricity,^[^ ^79^ ^]^ and piezoelectricity^[^ ^80^ ^]^ Transition of bandgap from indirect to direct was observed by Zhang et al. for TMD materials with thickness decreasing from bulk to monolayers.^[^ ^81^ ^]^ For instance, H‐phased MoSe_2_ has a bandgap of 1.41 eV for bulk and 1.58 eV for monolayer, close to theoretical Density‐functional theory (DFT) calculation result of 1.1 and 1.55 eV, respectively.^[^ ^82^ ^]^ The bandgap values of bulk and monolayer MoS_2_ was measured as 1.27 and 1.8 eV,^[^ ^83^ ^]^ with the reported DFT‐calculated values of 1.44 and 2.22 eV, respectively.^[^ ^84^ ^]^ Furthermore, a similar observation was reported with other H‐phased TMD materials.^[^ ^85^ ^]^

Another attractive physical property for the TMD layered material is the spin splitting attributed to both spin–orbit coupling and interlayer coupling. Missing inversion symmetry not only enables pin splitting at the edge of valence band,^[^ ^87^ ^]^ but also at conduction band, though much weaker.^[^ ^91^ ^]^ The effect of only spin–orbit coupling is suggested for monolayer MoS_2_, while both spin–orbit coupling and interlayer coupling play critical roles in the band structures for multilayered MoS_2_.^[^ ^92^ ^]^ As revealed from the theoretical study, splitting of conduction band minimum and valance band maximum is induced by the interlayer coupling and spin–orbit coupling separately. A trend of larger splitting for heavier metal elements was reported since the electrostatic interaction and relativistic effects critically impact spin–orbit coupling, which displays a positive relationship with band splitting. For instance, larger splitting from 0.15 to 0.46 eV was reported for 2H‐MoS_2_ and 2H‐WSe_2_, respectively.^[^ ^93^ ^]^ The spin–orbit coupling, valley polarization and representative non‐linear optics (NLO) are determined by the stacking order and subsequent structural symmetry.^[^ ^94^ ^]^

**Figure 4:**
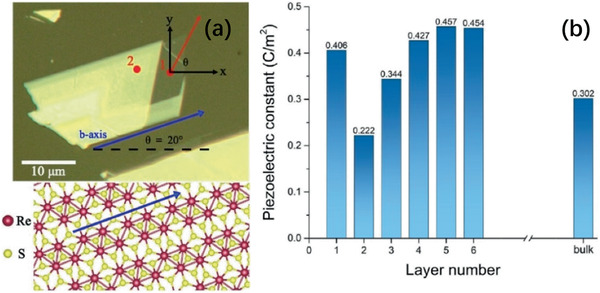
a) Microscope image and the distorted 1T lattice structure of ReS_2_. The red dots 1 and 2 are the laser focusing positions for monolayer and multilayer measurements, respectively. The blue arrow indicates the *b‐*axis direction, which is the direction of the Re atom chains in the lattice. Reproduced with permission.^[^ ^89^ ^]^ Copyright 2017, American Physical Society. b) Piezoelectric constant of 3R‐MoS_2_ as a function of the layer number computed at a fixed strain *ε*_11_ = −5%. Reproduced with permission.^[^ ^90^ ^]^ Copyright 2019, Elsevier.

Piezoelectricity pushes forward 2D TMDs in nanosensor and nanogenerator applications because of their anisotropic piezoelectric coefficient and power output.^[^ ^95^ ^]^ As reported by Kim et al., the piezoelectric coefficient of monolayer MoS_2_ in the armchair direction is 3.78 pm V^−1^, while that in the zigzag direction reaches 1.38 pm V^−1^, revealing its anisotropic piezoelectric property and providing a new way harvesting mechanical energy in low power‐consuming devices and self‐powered electronics.^[^ ^96^ ^]^ Stacking of 2H‐MoS_2_ with even number of layers will eliminate piezoelectricity for centrosymmetry.^[^ ^97^ ^]^ Broken inversion symmetry in odd‐layered 2H‐TMDs have the piezoelectricity as 1/N of the number of layer N.^[^ ^95^ ^]^ For phase 3R, the period stacking way breaks absence of inversion symmetry for both even and odd layered TMD, making the issue differently.^[^ ^86^ ^]^ Tan et al. has demonstrated that the 3R‐MoS_2_ with 5 layers has the strongest piezoelectricity (**Figure** **4**b).^[^ ^90^ ^]^ The piezoelectric effect of TMD refers to a complicated mixture of surface effects, electronic interactions and atomistic structure details that requires large scale self‐consistent numerical calculations.^[^ ^88^ ^]^

### 2.3 Electronic Properties of 2D MPTs

Gaining insights into the electronic properties of 2D MPT layers underpins the possible integration in nanodevices and applications.^[^ ^98^ ^]^ In layered MPT material, the interlayer coupling is the relatively weak van der Waals interactions, enabling thinning methods to the monolayer limit by exfoliation technique.^[^ ^99^ ^]^ Similar to TMDs, thinning of materials to monolayer leads to intrigue electronic properties for the number of electrons in the outmost shell or d orbital of the metal elements constituted in layered MPT. Besides, the [P_2_X_6_]^4−^ provides a weak ligand field, resulting in a high spin state of the metal atoms.^[^ ^100^ ^]^ Since metal phosphorus trichalcogenides are naturally layered structure, it is natural to anticipate that 2D MPT may exhibit prominent electronic properties compared with their corresponding bulk.

**Figure 5:**
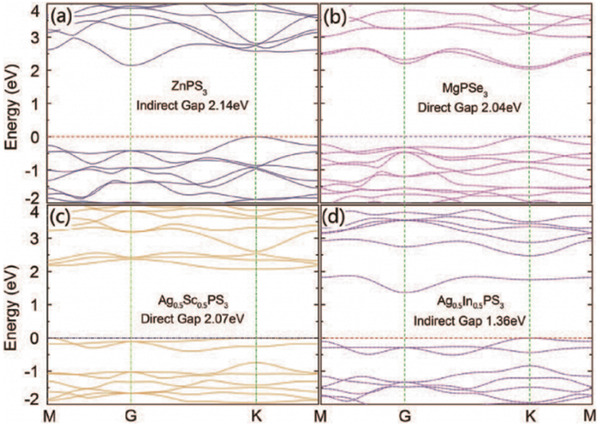
Electronic structure of monolayered a) ZnPS_3_, b) MgPSe_3_, c) Ag_0.5_Sc_0.5_PS_3_, and d) Ag_0.5_In_0.5_PS_3_ calculated with PBE functional. Reproduced with permission.^[^ ^101^ ^]^ Copyright 2014, AIP Publishing LLC.

With the thickness reduced to limit, monolayered MPT structure displays a representative structure of the layered MPT materials. Given the conduction band minimum (CBM) and valence band maximum (VBM) positions in the reciprocal space, the band edges of all the monolayered MPTs can fall to four categories .^[^ ^101^ ^]^ As given **Figure** **5**, monolayered MPT (M = Zn, Cd, and In, chalcogenide = S, Se) has a direct bandgap with the CBM is at Gamma (G) point and the VBM is at K point and Ag_0.5_In_0.5_PX_3_ (X = S and Se) is indirect gap semiconductors with the VBM and CBM located at an opposite position. Besides, MgPX_3_ and Ag_0.5_Sc_0.5_PX_3_ are indirect bandgap semiconductor. Edges of the valence band and conduction band comprise the bonding and the antibonding levels derived from the P—P bonds. Jenjeti et al. suggested that in the 2D NiPS_3_ material, the P—P is identified in the lower valance band (−5 to −7 eV) energy region, revealing the presence of large population of 3p orbital of S at the Fermi by orbital projected density of states (DOS) of individual atoms, whereas the contribution by phosphorus is negligible.^[^ ^102^ ^]^ First, Mercier et al. apply electronic structure calculated from the ionic extreme of the Wilson–Yoffe band model and extended Huckel mode to explain optical absorption spectra of MPS_3_, revealing semiconducting behavior of the mentioned compounds.^[^ ^103^ ^]^ Similar electronic bandstructure was reported for the mentioned MPTs with transition metal atoms from first raw series, compounds possess similar electronic band structures.

Metal atom critically impacts the bandgap. Moreover, bandgaps of selenides are smaller compared with those of sulfide because of relatively stronger electronegativity Se atoms. In 1996, Zhukov et al. demonstrated the relation between electronic structure and spin polarization in metal ions.^[^ ^104^ ^]^ The linear muffin‐tin method with the atomic sphere approximation was adopted to demonstrate the relative energies of t_2g_ and e_g_ bands. The DOS of MPTs give rise to the prediction of stability. The spin‐up states with lower energy are more stable as compared with the spin‐down states, thereby demonstrating a high‐spin configuration, which displays a tight relationship to magnetic properties.^[^ ^105^ ^]^

Conventionally, there are three types of magnetic moment distributions proposed for bulk MPT crystals. Type I can be expressed by the presence of double parallel ferromagnetic chains anti‐ferromagnetically coupled to each other (CoPS_3_, NiPS_3_). For type II layers, a magnetic ion is antiferromagnetically coupled with the three nearest neighbors in the layer, in which the net magnetic moments point to the basal planes (MnPS_3_) vertically. Specific to type III, the respective magnetic ion is ferromagnetically coupled with two of the three nearest neighbors within the single layer. In contrast to type I, each chain is antiferromagnetically coupled to the surrounding chain (FePS_3_). Study of bulk MPT materials has a long story, while the monolayered MPT is usually studies theoretically.^[^ ^106^ ^]^ For the MPT monolayer, there are four magnetic orderings, i.e., ferromagnetism (FM), Neel anti‐ferromagnetism (AFM), zigzag AFM (zAFM), and stripy AFM.^[^ ^53^, ^107^ ^]^ Chittari et al.^[^ ^98^ ^]^ reported that all the spin‐states of the metal atoms show the identical orientation by investigating FM ordering of single‐layer MPT via first‐principles calculations. Opposite‐oriented spin‐states was observed in the nearest neighbor metal atoms for AFM order. In addition, the opposite‐oriented zAFM ordering is unique with the spin‐state of the adjacent metal atom in an array along the zigzag direction. However, the spin‐state of the adjacent metal atoms for the stripy antiferromagnetic ordering chain along the armchair direction shows an opposite orientation. According to DFT calculation, AFM ordering is confirmed as the most stable state for monolayers MPTs (M = V, Mn, and Ni, chalcogen = S, Se, and Te). Moreover, the nonmagnetic ordering is more stable for monolayer MPTs (M = Co, Cu, Zn, and Fe).^[^ ^98^ ^]^ Besides, monolayered FePS_3_ prefers the zAFM ordering, and CrPS_3_ and CrPSe_3_ are ferromagnetic. The crystal structure of MPT family with 3d transition metal has aroused huge attention for antiferromagnetic (AF) ordering as a hint for significant electronic correlations.^[^ ^108^ ^]^ Bulk MPT materials are capable of displaying diverse AF structures (e.g., zigzag and stripy type). Suppressing order of superconductivity emerges in FePSe_3_, increasing *T*_c_ from 2.5 to 5.5 K with the external pressure from 9 to 30 GPa, bearing resemblance to high *T*_c_ cuprates and iron‐based superconductors.^[^ ^109^ ^]^ As indicated from all these accumulated evidences, electronic correlations may be critical to the family of TMPTs.

Consistent with TMD, the appearance of two magnon scattering and change of the Raman peak positions or intensities suggest ordered spin states in the Raman spectra of magnetic crystals.^[^ ^110^ ^]^ It is noteworthy that the changes in the Raman spectrum of AFM materials refer to good signals for monitoring their magnetic ordering with a magnetic transition. Wang et al. reported magnetic persistence in monolayer FePS_3_ nanosheets, which revealed that the intralayer spin arrangement dominates the magnetic structure.^[^ ^111^ ^]^ By monitoring the intensity of the Raman peaks (P1a) belonging to zone folding at *T*_c_, Lee et al. investigated AFM ordering of FePS_3_ nanosheets exhibiting different layers^[^ ^53^ ^]^ An Ising type AFM ordering was observed when thinning to the monolayer limit (0.7 nm). Moreover, *T*_c_ (≈118 K) remains not related to the thickness of FePS_3_, suggesting that the weak interlayer interaction slightly impacts the AFM ordering.

## 3 Synthetic Approaches of the 2D Phase Transition Materials

The exploration of the properties and tic applications of 2D phase transition materials is largely determined by the development of simple and reliable synthetic strategies. In the following section, the synthetic strategies and the progress of 2D phase transition materials are discussed.

### 3.1 Synthesis of TMDs

For the TMDs, i.e., the most common phase transition materials, the 2H phase TMDs usually exhibit semiconductor characteristics and apply to the application of optoelectronic devices. However, the 1T or 1T’ phase TMDs exhibit metal characteristics and may act as catalysis materials. For this reason, to achieve their catalytic applications, phase transition strategies primarily aim to obtain specific phase materials to satisfy the requirements of high‐performance catalytic applications. One of the strategies is phase conversion from H phase to T or vice versa by nanotechniques (e.g., alkali ion intercalation, electrostatic doping, stress induction, thermal treatment, and external irradiation). Another strategy obtaining target phase is facilitating phase selecting during synthesis processes including chemical vapor deposition (CVD), chemical vapor transport (CVT), molecular beam epitaxy (MBE), physical vapor deposition (PVD), and liquid‐phase method.

#### 3.1.1 Strategies of Phase Conversion for TMDs

##### Alkali Ion Intercalation

**Figure 6:**
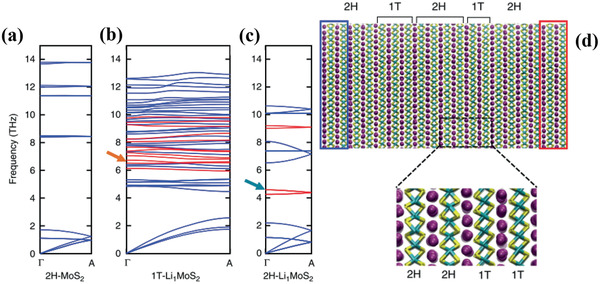
Calculated phonon dispersions along the cross‐plane of a) 2H‐MoS_2_, b) 1T‐Li1MoS_2_, and c) 2H‐Li_1_MoS_2_. Phonon branches are color‐coded based on whether they are MoS_2_‐like (blue) or Li‐like (red). d) Snapshot of a 10 nm thick NEMD simulation cell showing the mixed phase of Li_1_MoS_2_ system and a zoom‐in of a 2H‐1T phase boundary. Reproduced with permission.^[^ ^115^ ^]^ Copyright 2018, Nature Publishing Group.

Alkali ion intercalation is a hotspot in theory and experiments for phase conversion currently. 2D materials is a kind of layered materials with van der Waals forces between layers and relatively large layer spacing. Take TMDs as an example, alkali ions with small size (e.g., Li, Na, and K) thus can be easily inserted into layers of TMDs to form A*_x_*MX_2_ (A stands for alkali ions), causing charge doping and phase conversion.^[^ ^112^ ^]^ For MoS_2_, 2H‐MoS_2_ acts as a semiconductor with a hexagonal layered structure, and each Mo atom is connected to six S atoms. As impacted by lithium or sodium intercalation, the lattice of MoS_2_ matrix underwent a first‐order phase transition, and Mo coordination changed from a trigonal prismatic phase (2H structure) to an octahedral phase (1T structure).^[^ ^113^, ^114^ ^]^ As revealed from the calculations of the first principles conducted by Sood et al., the thermal and electrical conductivity modulation is enhanced by ion intercalation as a result of phonon scattering by lithium rattler modes, *c*‐axis strain, and stacking disorder (**Figure** **6**).^[^ ^115^ ^]^ As proved by the experimentally achieved results of Li intercalation into MoS_2_ and other materials, the in‐plane electrical conductivity is enhanced by two orders of magnitude. The phase conversion between 2H phase and 1T phase attributed to intercalation also exists in other TMDs. Modification of band filling state, Fermi level and perturbation in the phonon propagation introduced by alkali ion intercalation can effectively improve optical, thermal, and electrical properties. This makes the intercalated 2D materials suitable to be implemented in the functional optoelectronic devices as well as energy conversion applications (e.g., thermoelectrics and photovoltaics).

##### Electrostatic Doping

Electrostatic doping exploits an external electrostatic field to inject charge into the 2D materials to change the electron concentration of materials, so the phase conversion is likely to occur. Since the doping of external charge is controllable and reversible, this method is considered a nondestructive and reversible phase conversion strategy. In 2016, Reed et al. theorized that the change of chemical potential of electron or carrier density attributed to the applied gate voltage can cause the phase transition of single‐layered TMDs.^[^ ^116^ ^]^ In 2017, Zhang et al. realized the reversible phase transition of monolayer MoTe_2_ between 2H‐1T’ phase by electrostatic doping through applying and withdrawing gate voltage.^[^ ^117^ ^]^ In Zakhidov, Reed et al. demonstrated the phase transition of MoTe_2_ of all thickness from monolayer to bulk (73 nm) using ionic liquid‐based gate voltages at room temperature and ambient conditions.^[^ ^118^ ^]^ As revealed from the experimentally achieved results, the critical transition voltage increases with the increase in the thickness of MoTe_2_, demonstrating that a thicker sample requires a higher charge density. Also, Te atom vacancies generated during electrostatic doping have been proved as another vital reason for the phase transition of MoTe_2_. Liao et al. reported the Schottky barrier of metal/MoS_2_ interface and non‐overlapped channel region can be effectively tuned by electrostatically doping for MoS_2_ nanosheet (3.6 nm thick).^[^ ^119^ ^]^ The current density via the Schottky junction was illustrated to be proportional to the possibility of the carriers that overcome the interface barrier and move to active sites for catalytic reactions.^[^ ^120^ ^]^ In summary, the phase conversion induced by electrostatic doping uniformly occurs in the entire layered material, and the ionic liquid based gate voltages can reach a good doping level. Thus, electrostatic doping is considered to have broad applications in the dynamic phase conversion control of TMDs.

##### Stress Induction

**Figure 7:**
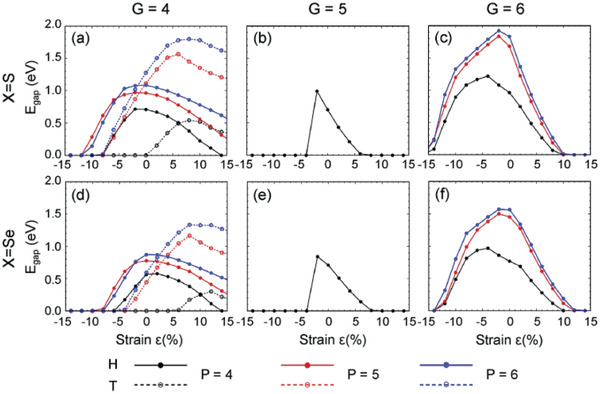
Electronic bandgap as function of strain in a) MS_2_(group 4), b) MS_2_(group 5), c) MS_2_(group 6), d) MSe_2_(group 4), e) MSe_2_(group 5), and f) MSe_2_(group 6). Reproduced with permission.^[^ ^125^ ^]^ Copyright 2019, Elsevier.

The stress induction method employs mechanical force to change the lattice structure of 2D materials and subsequently realizes the phase conversion. In 2014, Reed et al. showed that for most TMDs, the strain required to induce the phase transition is quite large, whereas MoTe_2_ only requires a tensile strain of less than 1.5% to achieve the phase transition, which enables it to induce the phase transition with strain.^[^ ^121^ ^]^ In 2016, Lee et al. exploited the stresses applied by the tip of a probe of AFM to enable MoTe_2_ to achieve a reversible phase transition from the 2H phase of semiconductor properties to the 1T’ phase of metal properties.^[^ ^122^ ^]^ Alternatively, the substrate stress is proved to be practically significant for the phase transition of TMDs.^[^ ^123^, ^124^ ^]^ Kang et al.^[^ ^125^ ^]^ reported that the resultant strain, either tensile or compressive, induce a structural phase transition by reducing the transition energy barrier, which also helps improve the catalytic performance (**Figure** **7**). Phase conversion through external stress indicates a preference for TMDs with metals in different groups. Phase transition for TMDs (M in group 4) from the equilibrium T phase to H counterpart is not preferred with external stress applied. H‐phased TMDs with group 5 metals can be converted into the T phase by external tensile stress but not vice versa. Besides, a spontaneous transition from phase H to T counterpart is induced by external stress on TMDs (M in group 6). Furthermore, the temperature significantly impacts the stress‐induced phase change, and the strain required for induced phase change decreases with the increase of temperature.

##### Thermal Treatment

Thermal treatment refers to another feasible method of inducing the phase conversion of 2D materials. In 2015, Lee et al. reported the phase conversion from 2H‐MoTe_2_ to 1T’‐MoTe_2_ in CVD system realized by rapid annealing.^[^ ^126^ ^]^ In 2015, as reported by Kim et al., under the temperature higher than 500 °C, the 2H‐MoTe_2_ obtained by flux method begin to be converted into the stable 1T’ phase. Further, 2H phase can be obtained by slow cooling from 900 °C to room temperature, and 1T phase can be obtained by quenching or rapid cooling.^[^ ^127^ ^]^ In 2018, according to Wang et al., the phase transition between 1T and 2H was identified on the surface of bulk‐TaS_2_ under the hot annealing treatment.^[^ ^128^ ^]^ Castelino et al.^[^ ^129^ ^]^ reported synthesis of pure 2H‐phased MoTe_2_ on SiC substrate under a substrate temperature of 250 °C during growth. While 1T‐phase is more stable for MoTe_2_ for most case. Interesting, pure 1T’ phased MoTe_2_ film with thickness of 35 nm was prepared under a higher temperature (450 °C), highlighting importance of temperature for phase transition.^[^ ^130^ ^]^ In brief, the thermal treatment method is recognized as an effective and reversible phase conversion induction method, whereas its difficulty lies in accurately controlling the temperature during the thermal treatment.

##### External Irradiation

External irradiation (e.g., plasma,^[^ ^131^ ^]^ electron beam,^[^ ^132^ ^]^ and laser^[^ ^133^ ^]^) provide other potential methods for inducing 2D materials phase transitions. The lattice reconstruction and partial vacancy in materials attributed to the high kinetic energy provided by external irradiation are considered the main reasons for the induced phase transition.^[^ ^134^, ^135^ ^]^ Zhu et al. reported an Ar‐plasma irradiation to induce the phase transition of monolayer MoS_2_ from 2H phase to 1T phase.^[^ ^136^ ^]^ In 2018, Tan et al. suggested that laser irradiation is capable of inducing the phase transition from 2H to 1T’ phase in few‐layered MoTe_2_, demonstrating the irreversible phase transition origins from the formation of Te vacancies due to laser local instantaneous heating.^[^ ^137^ ^]^ A reverse transition from 1T’ to 2H of MoTe_2_ has been reported by Nan et al. using soft hydrogen plasma.^[^ ^138^ ^]^ Furthermore, the external irradiation method can easily achieve the controlled phase transition in the target region due to controllability and programmability of plasma, electron beam, and laser. However, it is noteworthy that external irradiation will often cause some damage to the sample.

##### Others

Besides alkali ion intercalation, electrostatic doping, stress induction, thermal treatment, external irradiation, some other methods (e.g., interlayer coupling,^[^ ^139^ ^]^ chemical modification,^[^ ^140^ ^]^ and alloying^[^ ^141^, ^142^, ^143^ ^]^) can be adopted to achieve the phase transition of 2D materials. Notably, the possible electron transfer between the substrate and the materials also affects the structural stability of the 2D materials, thereby leading to phase transitions.^[^ ^144^, ^145^, ^146^ ^]^

#### 3.1.2 Strategies of Phase‐Selective Synthesis for TMDs

CVD, CVT, MBE, PVD, and liquid‐phase method refer to the major methods of directly synthesizing 2D materials with the target phase. For TMDs were mainly used, i.e., CVT,^[^ ^143^, ^147^ ^]^ CVD,^[^ ^148^, ^149^, ^150^, ^151^ ^]^ MBE,^[^ ^152^, ^153^, ^154^ ^]^ liquid‐phase method.^[^ ^142^ ^]^ Jiao et al. reported CVT deposition of 1T‐TiSe_2_, and charge density wave was observed in 5 nm TiSe_2_ nanosheets.^[^ ^147^ ^]^ Likewise, h‐BN was reported to as the substrate to synthesize monolayered 1T‐TaS_2_. Another report of obtaining W doped MoTe_2_ (i.e., Mo_1−_ *_x_*W*_x_*Te_2_) single crystal by CVT technology demonstrates the influence of W content on the phase of MoTe_2_.^[^ ^143^ ^]^ In the case of CVD, h‐BN was reported to be used as the substrate for synthesis of TMDs, such as monolayered 1T‐TiSe_2_ and NbSe_2_.^[^ ^150^ ^]^ Batzill et al. reported the deposition of a single layer 1T‐VSe_2_ on highly oriented pyrolytic graphene (HOPG) and MoS_2_ substrates with MBE method.^[^ ^154^ ^]^ Meanwhile, more routes to tune structure of the phase‐transition material have been explored. Ajayan et al. synthesized high‐quality rare earth element doped MoSe_2_. As indicated from their study, the preferred phase of the obtained MoSe_2_ was determined by the concentration of the doped rare earth atoms. Under the concentration lower than 40%, the 2H phase is more stable, otherwise, the 1T prime phase is more stable.^[^ ^148^ ^]^ Another phase‐control route is the Liquid‐phase method reported by Huang et al. to prepare few‐layer Mo*_x_*W_1−_ *_x_*S_2_ nanosheets. The concentration of 1T phase in the product was proved to be controlled by the reaction temperature.^[^ ^142^ ^]^

Specific to TMDs, CVD is the most promising method, whereas its difficulty is the need for accurate condition control and optimization, including precursor design, temperature control, atmosphere regulation, etc. Alkali ion intercalation is a widely studied method for inducing phase transition of TMDs. This process of inducing phase transition is relatively controllable and partially reversible. However, more theories and experiments are required to further understand the mechanism and process of intercalation induced phase transition. Electrostatic gating is promising because it is reversible and nondestructive, whereas the doping concentration and depth are relatively small, which should be further improved. The stress method has a broad prospect, and the stress threshold for phase transition can be adjusted by temperature. Thermal treatment needs special care for the phase transition engineering as it would inevitably introduce defects and often brings with damage in the material structure for high temperature process. External irradiation refers to a relatively clean method with programmable and controllable properties. However, high‐energy particles may damage samples, so external irradiation conditions should be further controlled and optimized. In summary, the preparation strategies of TMDs primarily include the following three challenges: i) Phase transitions are usually reversible. The TMDs of the metallic phase exhibit high conductivity and abundant reactivity sites, so they are an excellent electrical catalyst. However, the TMDs of the metallic phase usually display a metastable structure, so the phase transition from the semiconductor phase to the metallic phase is commonly reversible. ii) Some 2D TMDs are unstable as impacted by the influence of oxygen and water in the environment. Therefore, the subsequent stability of the target phase products should be considered in different phase transition strategies. iii) Phase purity is a vital factor of the catalytic performance of materials.

### 3.2 Synthesis of MPTs

The synthesis of MPTs includes the preparation of MPTs crystals and the preparation of 2D MPTs To be specific, the main method of preparing MPTs crystal is CVT method. The preparation methods of 2D MPTs mainly include CVD method, micromechanical exfoliation method, intercalation method, and ion‐exchange solvothermal method.

#### 3.2.1 CVT Method

**Figure 8:**
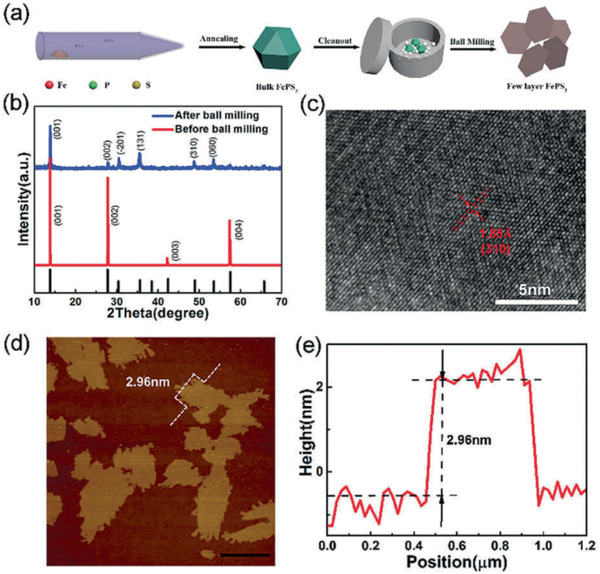
a) Synthesis procedure of few‐layered FePS_3_ nanosheets. b) X‐ray diffraction patterns of the as‐synthesized FePS_3_ before (red) and after (blue) ball milling. c) High‐resolution TEM (HRTEM) image of the FePS_3_ after ball milling. d) AFM image and e) line‐scan profile of the FePS_3_ after ball milling. Reproduced with permission.^[^ ^155^ ^]^ Copyright 2018, RSC.

The most common method for preparing MPTs crystals is CVT method, which exhibits a high material conversion efficiency. CVT techniques are commonly reported to synthesize MPTs crystals with sufficient size. The preparation of CVT can be summarized as follows. The metal or metal compounds, sulfur powder and phosphorus powder in the higher temperature region (T_2_) in the vial form steam are then transferred to the lower temperature region (T_1_) under the action of the transport carrier (e.g., iodine) to form MPTs crystal. In 2017, as reported by Pumera et al., the temperature and rate of change in temperature during the growth of CVT significantly impacted the quality of the obtained MPT_3_ crystals.^[^ ^54^ ^]^ Cheong et al. successfully grew FePS_3_ single crystal from pure Fe, P, and S powders with CVT method.^[^ ^53^ ^]^ Kloc et al. synthesized and exfoliated most of the MPS_3_ and MPSe_3_ single crystals, including FePS_3_, MnPS_3_, NiPS_3_, CdPS_3_, ZnPS_3_, FePSe_3_, and MnPSe_3_.^[^ ^66^ ^]^ Zhu et al. reported the preparation of 2D FePS_3_ layers with CVT methods and exfoliated by ball‐milling, as given in **Figure** **8**.^[^ ^155^ ^]^ Besides the practice of MPT crystals, the CVT method was proved to be useful for preparing MPT3 with few layers. Liang et al. first synthesized the corresponding metal hydroxide nanosheet precursor with hydrothermal method, subsequently mixed it with red phosphorus and sulfur powder at 520 °C, and finally prepared the few‐layered FePS_3_, CoPS_3_, and NiPS_3_ sheet with an average thickness of 18 nm.^[^ ^156^ ^]^

#### 3.2.2 CVD Method

**Figure 9:**
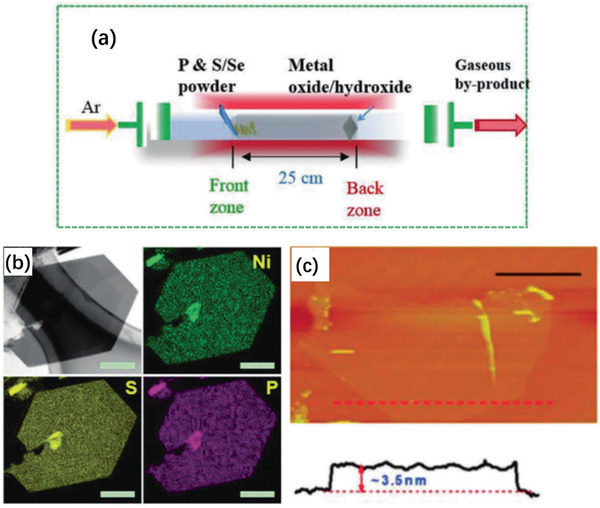
a) Schematic diagram of CVT method. Reproduced with permission.^[^ ^159^ ^]^ Copyright 2018, Wiley‐VCH. b) EDX elemental mapping and c) AFM image of ultrathin NiPS_3_ grown with CVD method. Reproduced with permission.^[^ ^158^ ^]^ Copyright 2017, Elsevier Ltd.

The CVD method has been commonly used for preparing 2D MPTs crystals with the prospect of mass production. Preparation of CVD can be summarized as follows: The reactants (e.g., sulfur powder and phosphorus powder) are first heated to the gas phase, and then deposited on the substrate (e.g., metal oxides and metal hydroxides) under the action of transport carrier (e.g., Ar_2_) and finally react to form the target 2D MPTs (**Figure** **9**a). The temperature during synthesis has been indicated to critically impact the formation of the product. For instance, the temperature of the reactant region should be carefully optimized to ensure that both the P and S/Se sources are vaporized; otherwise, an impure phase will be introduced. In 2017, as reported by Pumera et al., good CdPSe_3_ crystals can be synthesized only by performing long‐term low temperature synthesis (400–350 °C thermal gradient) and using iodine as a steam transport medium; otherwise, the CdSe always tends to be formed.^[^ ^54^, ^157^ ^]^ He et al. prepared 2D NiPS_3_ nanosheet with thickness ≤ 3.5 nm and lateral size > 15 µm by precisely controlling temperature and reaction time (Figure 9b,c).^[^ ^158^ ^]^ In 2018, they also grew MnPSe_3_ and MnPS_3_ nanosheets with high crystal quality with this method.^[^ ^159^ ^]^ In 2020, Liu et al. proposed a facile way to synthesize ultrathin FePS_3_, In_2/3_PS_3_, and CdPS_3_ nanosheets on fluorine‐doped tin oxide (FTO) substrates.^[^ ^160^ ^]^ For this reason, the careful optimization of reaction temperature gradient in CVD process is critical to obtain MPTs exhibiting high phase purity and high crystal quality. Moreover, the selected substrate is another important factor of product quality.

#### 3.2.3 Micromechanical Exfoliation Method

Since the success of the tape method for preparation of graphene, the micromechanical exfoliation method has been extensively employed for preparing 2D materials. The micromechanical exfoliation method is a method to obtain the corresponding 2D materials from the bulk materials via tape or force‐assisted liquid phase exfoliation.^[^ ^21^, ^161^ ^]^ As indicated from theoretical studies, the cleavage energy of most MPTs is smaller than that of graphite (≈0.36 J m^−2^), and their formation energy is significantly lower than that of single MoS_2_ (≈0.14 J m^−2^). Given the small cleavage energy and formation energy of MPTs materials, the bulk MPTs crystals are suggested to be easily exfoliated into 2D MPTs.^[^ ^66^, ^101^, ^162^ ^]^ The method of micromechanical exfoliation by tape can be recognized as a nondestructive technique since no other impurities are introduced in the exfoliation process. Thus, 2D MPTs samples obtained with this method have a clean surface and near‐perfect crystal quality. Besides, the lateral size of the 2D MPTs obtained with this method can reach tens of micrometer or even larger. The preparation of 2D MPTs with this method has been extensively reported,^[^ ^163^, ^164^, ^165^, ^166^ ^]^ whereas this method has low yield and high randomness, so it only applies to existing laboratory research. For the advantages of large‐scale controlled preparation, the micromechanical exfoliation method of liquid phase exfoliation has also aroused wide attention.^[^ ^167^ ^]^ Since the exfoliation environment covers various chemical solvents, and some alkali ions and surfactants may be added to facilitate the exfoliation, the 2D MPTs samples obtained with this method have considerable impurities and defects. However, the introduction of controllable impurities and defects is considered to be able to improve the catalytic activity of 2D MPTs effectively.

#### 3.2.4 Other Methods

Other methods with scarcely gas emission (e.g., intercalation^[^ ^67^, ^168^ ^]^ and electrochemical^[^ ^169^, ^170^, ^171^ ^]^) have been considered a green choice to prepare 2D MPTs. Alkali metals and organic molecules can be inserted into the interlayer of MPTs, thereby increasing the interlayer spacing and reducing the interlayer van der Waals forces. Accordingly, the intercalation method combined with the micromechanical exfoliation method may effectively improve the exfoliation efficiency of layered MPTs. Also, electrochemical process has been reported to prepare few‐layered NiPS3 sheet successfully.

For MPTs, the preparation methods consist of CVT method, CVD method, and micromechanical exfoliation, etc. The CVT method should precisely control the reaction conditions, significantly impacting the conversion efficiency and product type. It can be predicted that CVD method is a promising direction. But compared with TMDCs, MPTs belong to ternary compounds, the preparation is more difficult to control. Thus, CVD method will face many challenges. Some MPTs have been successfully prepared with CVD method, but compared with TMDs, the controlled growth of MPTs with monolayer, large size and high uniformity are more complicated. The main challenge of liquid phase exfoliation in the micromechanical exfoliation method is the control of introduced impurities and defects. Few MPTs based on the heavier chalcogen were reported, which requires creative research. Finally, the exploration of nonlayered 2D MPTs is another promising direction.

The strategies for preparing TMDs and MPTs display a tight relationship to the catalytic properties of the materials. The combination of other means to effectively regulate phase transition materials' catalytic properties refers to an important direction of future development. To be specific, the following aspects should be addressed: i) forming vdW heterojunction with other 2D materials, ii) an external magnetic field applied to improve the carrier mobility and catalytic performance of the materials, iii) metal doping or mixed alloy phase that can regulate catalytic performance, and iv) self‐assembled composite system with other nanostructures. v) On the one hand, advanced microscopic techniques and spectroscopic methods are considered capable of studying catalytic processes at the atomic scale and to building intelligent catalytic systems. On the other hand, the occurrence and reversal of the phase transition can be controlled by regulating the external conditions (e.g., the rise and fall of temperature or the application and release of stress) to control the catalytic reaction intelligently.

## 4 Catalytic Applications for 2D Phase Transition TMDs and MPTs

The topic of exploiting clean energy source instead of fossil fuels is arousing global attention. Hydrogen, as a green fuel source, however, can be used to directly convert chemical energy into electricity in fuel cells rather than combustion devices. As opposed to steam reforming that causes CO_2_ release, obtaining H_2_ by electrochemical hydrogen evolution reaction (HER) does not involve greenhouse gas emission, so it is relatively clean. HER or the cathodic half of water splitting reaction is written below

(1) $$2H_{3}O+2e^{-}\;⇌\;H_{2}+2H_{2}O$$

Generated electrons combine with protons on reactive sites, which are usually provided by catalysts, say Pt as an example. Moreover, conductive Pt catalyst contributes to fast transfer of electrons and holes, thereby accelerating the redox reaction. Though Pt exhibits the highest HER catalytic efficiency, high cost limits the use and stresses the importance of more semiconductor catalysts. Research publications have been increasing exponentially over the past decades. As the main process to obtain hydrogen from splitting H_2_O atoms via chemical/optical process, catalyst is the bottleneck of efficiency development thus far.

Research into 2D phase transition materials (e.g., TMDs and MPTs) highlights the critical impact of active sites on basal plane edges during catalytic HER reactions. Group‐VIB compounds are the most studied TMDs and the most potential candidates for the electrochemical hydrogen evolution reaction (HER). The phase transition from H to T‘ increases the number of active sites on the basal plane besides those on the edge sites. Moreover, thus activating basal plane and phase transition have been demonstrated to be effective methods for increasing catalytic efficiency. Thus, activating basal plane and phase transition are essential to catalyst and call the potential of 2D phase transition materials as catalysts.

Large surface areas of TMDs and MPTs (2D materials) provide surface area for mass exchange. Phase transition properties of TMDs and MPTs endow them with tunable electronic properties and surface functionalization. Moreover, photo‐driven catalytic reaction, exploiting light energy instead of electricity, stresses the significance of bandgap for harvesting light. 2D TMD and MPT materials have bandgaps in the visible light region, as demonstrated in photocatalytic reactions. For instance, water‐splitting, exhibiting the excitation energy of 2.13 eV, can be accelerated by TMD photocatalysts.

High electron mobility, conductivity, proper bandgap, and large surface area endow 2D phase transition TMDs and MPTs intrigue properties among catalysts. Besides, nanotechnologies that fine the size, shape, composition, structure, and design is critical to 2D phase transition materials to achieve higher efficiency in chemical/photocatalytic reaction (e.g., hydrogen evolution reaction (HER), oxygen evolution reaction (OER), oxygen reduction reaction (ORR), as well as carbon dioxide reduction reaction (CO_2_RR).

### 4.1 TMDs Catalysts

**Figure 10:**
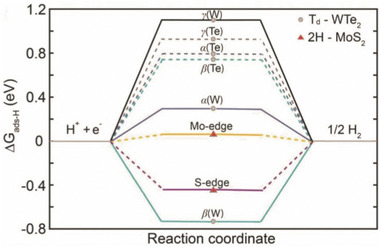
Comparison of the ∆*G*_H_ values between Td–WTe_2_ and 2H­MoS_2_ at various hydrogen­bonding sites. Reproduced with permission.^[^ ^173^ ^]^ Copyright 2016, John Wiley & Sons.

2D phase transition materials possess large surface area ratio and high electron mobility, paving the way to electrocatalysts in various electrochemical reactions. TMDs with tunable electrical properties have been proved to be active materials in various electrical applications, including HER, CO_2_RR, and water splitting, etc. Theoretical and experimental results demonstrated that regardless of the sample size, the catalytic reaction rate is proportional to the number of active sites.^[^ ^172^ ^]^ The TMD catalyst has the H‐atom activation energy, marked as ∆*G*_H_, decreases significantly from the *γ*(W) site (the basal plane site) to the *α*(W) site (the edge site).^[^ ^173^ ^]^ The ∆*G*_H_ values for the edge sites in WTe_2_ and MoS_2_ are given in **Figure** **10** for comparison.

A ∆*G*_H_ close to 0 is promoted for catalytic reaction and sites on the basal plane are relatively inactive compared to the edge sites. While the basal plane is the dominant thermodynamic stable exposure surface for TMDs, activating the basal plane is significant for improving the density of active sites.^[^ ^174^ ^]^ Alternative strategies are to activate the basal plane through nanoengineering, including introducing chalcogenide vacancies,^[^ ^175^, ^176^ ^]^ active metal ions,^[^ ^177^, ^178^ ^]^ charge doping,^[^ ^179^ ^]^ or dopant.^[^ ^180^, ^181^ ^]^

**Figure 11:**
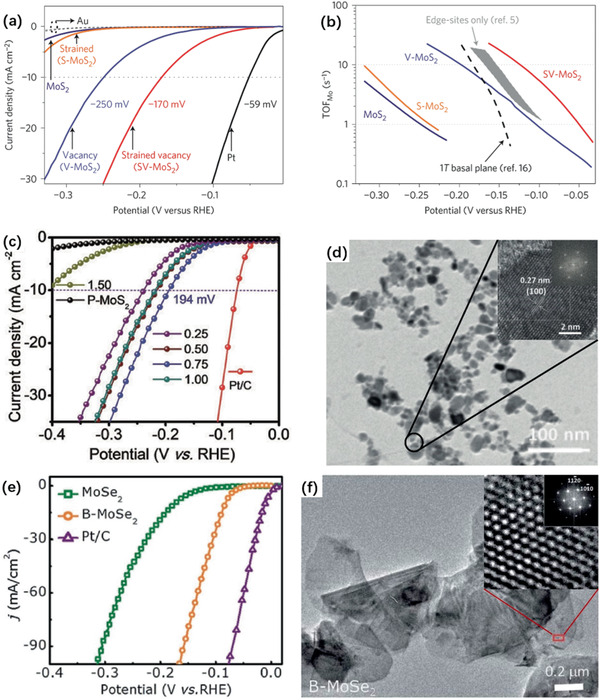
a) LSV curves for the Au substrate, Pt electrode, as‐transferred MoS_2_, strained MoS_2_ without S‐vacancies, unstrained MoS_2_ with S‐vacancies, and strained MoS_2_ with S‐vacancies. b) Corresponding Tafel plots of the LSV curves. a) and b) Reproduced with permission.^[^ ^175^ ^]^ Copyright 2016, Nature Publishing Group. c) Polarization curves of P‐MoS_2_ and Sv‐MoS_2_ via Zn reduction with increasing mass ratios zinc/MoS_2_. d) TEM and HRTEM images of Zn‐treated MoS_2_ nanosheets c,d) Reproduced with permission.^[^ ^177^ ^]^ Copyright 2019, John Wiley & Sons. e) The LSV curves of doped MoSe_2_ and B doped MoSe_2_ compared with a Pt/C electrode. f) TEM and HRTEM images of B‐doped MoSe_2_ nanosheets. e,f) Reproduced with permission.^[^ ^180^ ^]^ Copyright 2018, RSC.

Indicators, including the overpotential of 10 mA cm^−2^ have been applied for evaluating HER efficiency. Tafel slope is used as another indicator which monitors reaction progress and aids to decode the rate‐determining steps. Li and Tsai et al. discovered basal plane activation by creating S vacancies and straining on monolayer H‐MoS_2_ through electrochemical desulfurization.^[^ ^175^, ^176^ ^]^ With the combination of S‐vacancies and strains, the basal plane is promoted with a low overpotential of −170 mV versus RHE and a much higher TOF_S‐vacancy_ (0.08–0.31 s^−1^) of the MoS_2_ edge sites (at 0 V vs RHE), as given in **Figure** **11**a,b. Wu et al. reported enhanced catalytic efficiency with an overpotential of −194 mV at 10 mA cm^−2^ and a low Tafel slope at 78 mV dec^−1^ by activating MoS_2_ basal plane using zinc, as in Figure 11c,d. A high density of S‐vacancies, which are unfavorable by DFT, easily formed around the Zn atoms in MoS_2_ nanosheets_._^[^ ^177^ ^]^ The modulation effect of extra metal deposited on the basal plane of TMD in the catalytic reaction also applies for Pt,^[^ ^182^ ^]^ Ni,^[^ ^178^ ^]^ Cu,^[^ ^183^ ^]^ Co,^[^ ^184^ ^]^ etc. An alternative method to activating the basal plane is dopant, such as B,^[^ ^180^ ^]^ P,^[^ ^181^ ^]^ etc. Gao et al. demonstrated that B dopants in MoSe_2_ nanoflakes induce hybridization among Mo 3d, S 2p, and B 2p orbitals, creating more gap states, narrowing the bandgap increasing conductivity.^[^ ^180^ ^]^ The B dopant increases the 2D MoSe_2_ catalytic efficiency with a low overpotential (84 mV vs reversible hydrogen electrode (RHE)) and Tafel slope (39 mV s^−1^), as in Figure 11e,f.

Another method to enhance the catalytic activity is to improve the conductivity intrinsically. For group VI TMDs, the nature of basal planes are demonstrated to change from semiconducting to metallic with the phase transfer from 2H to 1T/T’.^[^ ^75^ ^]^ The conductive 1T/T’ phase of VI group TMD make it possible to have active sites on both edges of the basal plane and the layered grains. Because the charge transfer resistance is reduced in the 1T/1T’ metallic phase.^[^ ^185^ ^]^ As Norskov et al. first demonstrated, a smaller ∆*G* is used to predict higher catalytic property.^[^ ^186^ ^]^ In another aspect, reactant atoms on the basal plane with proper energy benefit the reaction rate. Considering the free energy of H atom on the basal plane, MoS_2_ in T phase has a lower ∆*G*_H_ (0.06 eV) compared to H‐MoS2 (2 eV) theoretical and experimental demonstrating by Hinnemann et al.^[^ ^187^ ^]^ and Jaramillo et al.^[^ ^72^ ^]^ respectively. The T‐phased MoS_2_ have a higher catalytic property than the H‐phased MoS_2_. While most of T‐phased group‐VI TMD materials, including MoS_2_, MoSe_2_, WS_2_, WSe_2_, etc., are not stable under an ambient electrocatalyst condition and has the trend of transforming into H‐phase.^[^ ^188^ ^]^ Meanwhile, for group IV, VII, and VIII TMD, phase transition have been theoretical and experimental demonstrated to create more active sites, thus benefit electrochemical catalytic reaction.^[^ ^189^ ^]^

For these reasons, various methods^[^ ^193^ ^]^ have been developed for TMD material phase transition in the past decade, including chemical exfoliation,^[^ ^194^ ^]^ mediating,^[^ ^195^ ^]^ interlayer coupling, charge doping, or chemical evaporation deposition.^[^ ^196^ ^]^

**Figure 12:**
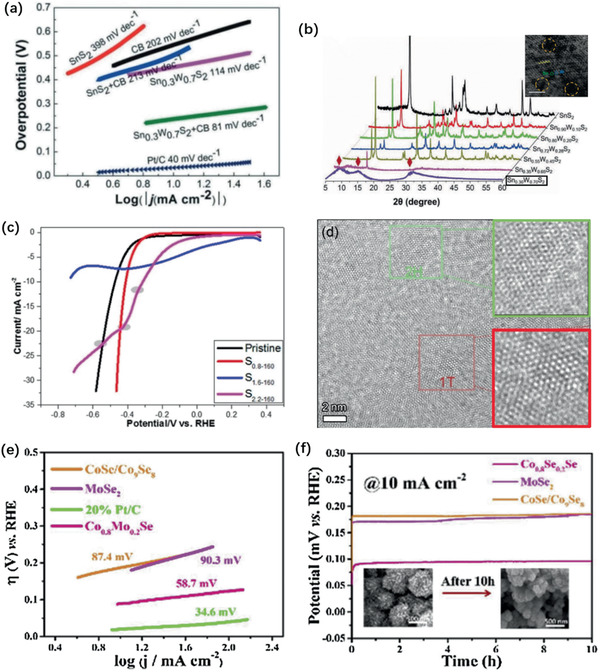
a) Cathodic Tafel slopes (over‐potential vs log│current density│) of Sn_1−_ *_x_*W*_x_*S_2_ nanosheet and Pt catalyst. b) XRD data of Sn_1−_ *_x_*W*_x_*S_2_ alloys and STEM image of metallic 1T′‐Sn_0.3_W_0.7_S_2_. (Yellow dotted line circles with some defects. Scale bar: 2 nm). a,b) Reproduced with permission.^[^ ^190^ ^]^ Copyright 2020, John Wiley & Sons. c) Polarization curves of the MoS_2_ nanosheets after electrochemical treatments with CVs. d) TEM images of MoS2 nanosheets desquamated from 1 T‐MoS_2_/Carbon Cloth. c,d) Reproduced with permission.^[^ ^191^ ^]^ Copyright 2018, ACS. e) Tafel plots for CoSe/Co_9_Se_8_, Co_0.8_Mo_0.2_Se, MoSe_2_ and 20% Pt/C in 0.5 m H_2_SO_4_. f) VT curves of CoSe/Co_9_Se_8_, Co_0.8_Mo_0.2_Se, MoSe_2_ at current density of 10 mA cm^−2^. e,f) Reproduced with permission.^[^ ^192^ ^]^ Copyright 2019, Elsevier B.V.

Initially, Lukowski reported transfer 2H‐MoS_2_ nanosheet into 1T phase through chemical exfoliation significantly promote the catalytic activity.^[^ ^194^ ^]^ The mixture of 1T and 2H MoS_2_ gave a clue to improve the electrochemical catalytic activity, characterized by a low overpotential of −187 mV versus RHE and a Tafel slope of 43 mV dec^−1^. A 2H‐1T’ phase transition of Sn_1−_ *_x_*W*_x_*S_2_ nanosheet was reported by Shao et al.^[^ ^190^ ^]^ Best among the gradient content nanosheets was Sn_0.3_W_0.7_S_2_ with lattice distortion displayed 81% metallic phase with an enhanced HER activity with an onset potential of 158 mV and Tafel slope 81 mV dec^−1^, as in **Figure** **12**a,b. Simple phase transition induced by vacancy has been reported by Gan et al. using electrochemical etching, with polarization curves given in Figure 12c,d.^[^ ^191^ ^]^ These results demonstrated that the S‐vacancies reduced the bandgap and decreased ∆*G*_H_ and stabilized the 1T phase by occupying Mo 4d orbital. Phase transition in CoSe instigated by Mo doping was reported by Zhou et al. to show a catalytic performance with an overpotential of 186.1 mV and Tafel slope of 58.7 mV dec^−1^, as in Figure 12e,f.^[^ ^192^ ^]^ Similar enhancement effects were reported for other transition metal dopants, including Fe,^[^ ^192^ ^]^ Zn,^[^ ^197^ ^]^ Ni,^[^ ^198^ ^]^ N,^[^ ^199^ ^]^ P,^[^ ^200^ ^]^ S,^[^ ^200^ ^]^ etc. While attempts to promote the catalytic efficiency with the doping phase transition method also has been reported failed, for instance, Nb/Ta doped MoS_2_ and WS_2_ reported by Chua et al.^[^ ^201^ ^]^

Phase transition trials of obtaining T‐phased TMD usually ends up in a mixture of these two phases.^[^ ^202^, ^203^ ^]^ Moreover, a reversal phase transition from 1T‐2H were observed in many cases.^[^ ^204^ ^]^ A recent study on activation basal plane by domain boundary was reported by Zhu et al.^[^ ^205^ ^]^ High catalytic efficiency was achieved by 2H‐2H and 2H‐1T boundary, showing remarkable catalytic performance with a small overpotential of ≈100 mV and Tafel slope of mV dec^−1^. Chen et al. demonstrated new categories appear in stable phase boundaries, including Mo, Te, and hollow sites, in the case of MoTe_2_ mixed‐phased catalyst in HER.^[^ ^73^ ^]^ Though the trials meaningful, there remains effort obtaining electro‐chemical stabled T‐phase TMD catalyst and controlling phase transition of TMD catalysts. Those TMD with stable 1T phase, high cost inhibits their application on a large scale.^[^ ^206^ ^]^ Appling cocatalyst in the catalytic system brings up to high conductivity with cost control. From graphene and C_3_N_4_ to carbon fiber or porous carbon, the exploration of carbon‐rich matrix has paved the way to cocatalyst with high conductivity and low cost. High conductivity of 1T/T’‐phased TMD also makes them an ideal component in cocatalyst. Some of the cocatalyst with TMD is summarized in **Table** **1**.

**Table 1:** Summary of HER performance of some 2D TMD catalysts

Catalyst	Main Phase	Average thickness [nm]	Loading [mg cm^−2^]	Overpotential [mV]	Exchange current density [mA cm^−2^]	Tafel slope [mV dec^−1^]	Stability	Ref.
Co/MoS_2_	2H	N/A	0.27	*η*_10_ = 185	68	200	11 h	^[^ ^207^ ^]^
MoS_2_@carbon fiber	1T	≈10.0	17	*η*_10_ = 151	N/A	55	1000 cycles	^[^ ^191^ ^]^
MoS_2_@C_3_N_4_	1T/1T’	N/A	0.28	*η*_10_ = 215	80	50.2	N/A	^[^ ^208^ ^]^
MoS_2_@ porous C	2H	15.0	1.00	*η*_10_ = 136	76	99	24 h	^[^ ^195^ ^]^
MoS_2_/Co(OH)_2_	1T’	8.2	0.2	*η*_10_ = 89	72.9	53	20 h	^[^ ^209^ ^]^
MoS_2_@Au	2H/1T	1.9	N/A	*η*_10_ = 136	57 × 10^−3^	73	200 h	^[^ ^205^ ^]^
CoS_2_@carbon cloth	2H	N/A	0.92	*η*_10_ = 112	N/A	60.1	N/A	^[^ ^210^ ^]^
TaS_2_@Au	2H	5.0	N/A	*η*_10_ = 101	N/A	53	12 h	^[^ ^211^ ^]^
TaS_2_@Au	1T	18–24	N/A	*η*_10_ = 207	67.61	67	24 h	^[^ ^212^ ^]^
WS_2_	1T	4 layers	0.10	*η*_10_ = 118	−21	43	30 h	^[^ ^213^ ^]^
FeS_2_/CoS_2_	2H	1.6	0.2	*η*_10_ = 78.2	N/A	302	80 h	^[^ ^214^ ^]^
Pd*_x_*NbS_2_	2H	2.0	0.25	*η*_10_ = 157	N/A	50	12 h	^[^ ^215^ ^]^
Ni_3_S_2_/FeS/CoS	1T	12.0	N/A	*η*_10_ = 170	N/A	68	50 h	^[^ ^216^ ^]^
MoSe_2_/Ti	1T	N/A	0.16	*η*_20_ = −133	121	68	20 h	^[^ ^217^ ^]^
N‐MoSe_2_	2H	N/A	N/A	*η*_20_ = −135	108.4	62	1000 cycles	^[^ ^218^ ^]^
MoS_2_/MoSe_2_/graphene	2H	3.0–5.0	0.12	*η*_20_ = 70	130	61	1000 cycles	^[^ ^219^ ^]^
TiO_2_/Si/MoSe_2_	2H/1T	N/A	N/A	*η*_10_ = −94	N/A	43	10 h	^[^ ^220^ ^]^
WSe_2_@carbon paper	1T’	N/A	0.04	*η*_10_ = 300	N/A	150	N/A	^[^ ^221^ ^]^
WSe_2_/Sn	1T	N/A	N/A	*η*_10_ = −86.6	N/A	36	5000 cycles	^[^ ^222^ ^]^
ReSe_2_@SiO_2_/Si	1T	0.73	N/A	*η*_10_ = 270	10.5 × 10^−3^	76	1000 cycles	^[^ ^223^ ^]^
ReSe_2_	1T	8–10	N/A	*η*_10_ = 265	N/A	69	2.7 h	^[^ ^224^ ^]^
FeNiSe@graphene	1T	30	3.3	*η*_10_ = −187	N/A	65	10 h	^[^ ^225^ ^]^
Ni_0.89_Co_0.11_Se_2_@Ni	1T	12	2.2	Η_‐10_ = 85	N/A	52	30 h	^[^ ^226^ ^]^
MoTe_2_@carbon cloth	1T’	N/A	N/A	*η*_10_ = −230.7	N/A	127.1	1000 cycles	^[^ ^227^ ^]^
MoTe_2_	1T’	≈2	N/A	*η*_10_ = 356	2.1 × 10^−2^	22	2000 cycles	^[^ ^228^ ^]^
TiP2S6@MoTe2	1T	≈20	13	*η*_10_ = 144	N/A	53	4.2 h	^[^ ^229^ ^]^
NiTe2@Ti mesh	1T	3–4 × 10^3^	0.95	*η*_10_ = 315	N/A	82	24 h	^[^ ^230^ ^]^
CoTe_2_@carbon paper	CoTe	N/A	4.85	*η*_100_ = 230	9.95 × 10^−3^	57.1	5000 cycles	^[^ ^231^ ^]^

The OER with sluggish reaction kinetic requires large overpotential and more electric energy, which is not favored in the cathodic hydrogen production and sustainable development.^[^ ^232^ ^]^ In this case, OER has demonstrated to be the rate‐limiting step in the water‐splitting procedure, emphasizing the importance of pursuing appropriate catalysts to significantly improve the reaction kinetics and reduce the overpotential of OER. The attractive Pt‐based catalyst for ORR and other reactions are not favored in OER because of microscopic reversibility only holds for an equilibrium process.^[^ ^233^ ^]^ Metal, including Pt, suffers oxidation in the cathodic reactions in OER thus presents surface property change, which is avoided in ORR reactions. Traditional OER electrocatalysts are usually noble metal oxides (IrO_2_ or RuO_2_). As a series of novel catalyst, 2D phase transition TMD materials attract interests in OER for the nature of modulating the electronic structure and flexible surface‐active sites.

Free energy adsorption was used as an indicator for the catalytic efficiency. The ideal electrocatalysts for the OER should not interact too strongly nor too weakly with the OER intermediates. Initially, Zhao et al. has computed monitored adsorption free energy and overpotential of TMD in OER, demonstrating the small difference between 3R‐phase TMD and 2H‐phase.^[^ ^234^ ^]^ For MTe_2_ which bind *OH too strongly, *OH protonation process is so tricky that OER activity is limited. In contrast, for MS_2_ and MSe_2_ which bind OH too weakly, the Δ*G*_OOH_ is weak due to the linear relationship, and thus Δ*G*_OOH_ is hard to adsorb on the substrate. For this reason, strategies decreasing MTe_2_ or increasing MS_2_ and MSe_2_ help promote the OER catalytic activity.

**Figure 13:**
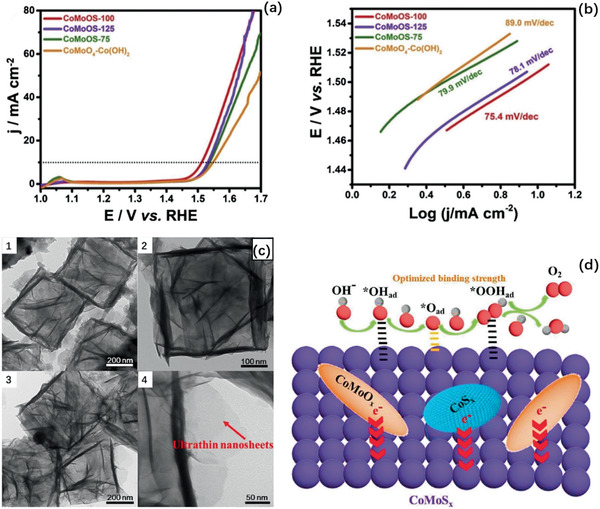
a) OER polarization curves, b) Tafel slope, and c) TEM of CoMoO_4_‐Co(OH)_2_, CoMoOS‐75, CoMoOS‐100, CoMoOS‐125 catalysts in OER. d) Scheme of OER process in CoMoOS NBs heterostructures. Reproduced with permission.^[^ ^235^ ^]^ Copyright 2020, Elsevier Ltd.

Activating the basal plane by various method (only doping^[^ ^216^ ^]^) paves the way of TMD into OER catalyst. The scalable technique provides an inspiring route for activation basal plane. As a key factor for catalyst, the surface area also acts as an advantage for 2D TMD material used in OER. Xu et al.^[^ ^235^ ^]^ reported 2D CoMoO*_x_*/CoMoS*_x_*/CoS*_x_* nanostructures used in the OER with an overpotential 281 mV at 10 mA cm^−2^ and Tafel slope of 75.4 mV dec^−1^ for CoMoOS‐100 NF//Pt/C NF, as in **Figure** **13**. The box structure built by ultrathin nanosheet brings up to a high electrochemical surface area and optimized biding energy of intermediate O*, thus facilitating the formation of OOH* and generation of O_2_. More uses of layered TMD materials as catalysts in electrochemical OER are given in **Table** **2**.

**Table 2:** Summary of OER performance of some 2D TMD catalysts

Catalyst	Main Phase	Electrolyte	Average thickness [nm]	Loading [mg cm^−2^]	Overpotential [mV]	Exchange current density [mA cm^−2^]	Tafel slope [mV dec^−1^]	Stability	Ref.
Co/MoS_2_	2H	1.0 м KOH	12.0	2.00	*η*_10_ = 260	N/A	85	3000 cycles	^[^ ^236^ ^]^
MoSSe/MWCNT	2H/1T	0.1 м KOH	3.6	N/A	*η*_10_ = 102	1.39	38	1 h	^[^ ^237^ ^]^
Fe/MoS_2_	1T	1.0 м KOH	N/A	N/A	Η_50_ = 328	N/A	126	8 h	^[^ ^238^ ^]^
ReS_2_	1T	0.1 м H_2_SO_4_	1.6	0.08	*η*_10_ = −68.89	N/A	141.9	6000 cycles	^[^ ^239^ ^]^
Pt@CoS_2_	2H	0.1 м KOH	N/A	0.50	*η*_10_ = 300	N/A	58	5000 cycles	^[^ ^240^ ^]^
Fe/CoS_2_	1T	1.0 м KOH	N/A	N/A	*η*_10_ = 230	N/A	128	20 h	^[^ ^241^ ^]^
Cu*_x_*Co_1−_ *_x_*S_2_/CNT	1T	1.0 м KOH	N/A	0.46	*η*_10_ = 284	N/A	86	8 h	^[^ ^242^ ^]^
NbS_2_@carbon paper	2H	1.0 м KOH	N/A	0.29	*η*_10_ = 178	N/A	278	10 h	^[^ ^243^ ^]^
MoSe_2_@rGO	2H	0.1 м KOH	N/A	0.29	*η*_5_ = 250	N/A	62	6 h	^[^ ^244^ ^]^
Ni_0.35_Co_0.65_Se_2_@Cu	1T	1.0 м KOH	20	N/A	*η*_10_ = 193	N/A	58	100 h	^[^ ^245^ ^]^
CoTe_2_@Ni	1T	1.0 м KOH	2–5	1.24	*η*_10_ = 310	N/A	54	27 h	^[^ ^246^ ^]^
NiTe_2_/Ni(OH)_2_	1T	1.0 м KOH	N/A	1.38	*η*_10_ = 267	N/A	67	6 h	^[^ ^247^ ^]^

Inspired by the electrochemical efforts, several inorganic catalysts including semiconducting metal oxides have been used for photocatalytic water‐splitting. Robust and stability in a photocorrosion environment make semiconducting metal oxide nanoparticles preferred photocatalyst. However, a relatively high positive valence band (O 2p) of most metal oxides hindered the potential because both sufficient negative conduction band to reduce H_2_ and a small bandgap is required to benefit the catalytic efficiency. Alternatively, TMD 2D materials with less positive valence bands and stability have been attracting interests as photocatalysts in water splitting. The phase transition from semiconductor into conductor or vice versa contributes to designing hetero‐catalysts' various strategies during a photo‐induced reaction. Some examples are listed in **Table** **3**.

**Table 3:** Summary of photoinduced water‐splitting of some 2D TMD catalysts

Catalyst	Main Phase	Thickness [nm]	Loading [mg mL^−1^]	Bandgap [eV]	sacrificial agent	Hydrogen evolution rate [mmol g^−1 ^h^−1^]	Quantum efficiency	Stability	Ref
MoS_2_	1T/1T’	1–10	0.25	N/A	15% triethanolamine	≈8	N/A	7 h	^[^ ^258^ ^]^
MoS_2_/g‐C_3_N_4_	1T	2.6	0.5	1.3–1.9	10% triethanolamine	0.51	N/A	N/A	^[^ ^259^ ^]^
MoS_2_/N‐doped Graphene	1T	N/A	0.05	N/A	15% triethanolamine	0.83	N/A	10 h	^[^ ^260^ ^]^
MoS_2_/pyrene	2H	1–10	0.5	N/A	10% methanol	0.12	N/A	20 h	^[^ ^261^ ^]^
MoS_2_/CdS	2H	4–10	0.625	2.12	0.5 m Na_2_S/Na_2_SO_3_	1.31	30.2%	18 h	^[^ ^262^ ^]^
MoS_2_/CdS	2H	Few layers	N/A	1.64	ethanol	140	66%	150 h	^[^ ^263^ ^]^
SnS_2_	2T	22	0.2	2.08	0.1 m Na_2_S/Na_2_SO_3_	1.06	N/A	6 h	^[^ ^264^ ^]^
SnS_2_/TiO_2_	2H	10	0.5	2.5	50% methanol	0.65	N/A	N/A	^[^ ^265^ ^]^
WS_2_/TiO_2_	2H	≈2	0.2	2.0	Sacrificial agent free	0.23	N/A	53 h	^[^ ^266^ ^]^
WS_2_/CdS	2H	3–7 layers	0.07	N/A	20% lactic acid	185.79	40.5%	50 h	^[^ ^267^ ^]^
NiS_2_/Fe/CdS	2H	10	1	N/A	10% methanol	3.2	N/A	18.3 h	^[^ ^268^ ^]^
MoSe_2_/SnSe_2_	2H	N/A	N/A	0.61	Sacrificial agent free	N/A	10.5%	N/A	^[^ ^269^ ^]^
MoSe_2_/Si	2H/1T	N/A	0.04	3.65	10% triethanolamine	167.6	N/A	10 h	^[^ ^220^ ^]^
MoSe_2_	1T	Few layers	N/A	N/A	15% triethanolamine	0.06	N/A	30 h	^[^ ^270^ ^]^
MoSe_2_/eosin Y	1T	≈1	0.05	N/A	20% triethanolamine	62	N/A	20 h	^[^ ^271^ ^]^
MoSe_2_/C_3_N_4_	2H/1T	≈1	0.1	N/A	10% triethanolamine	1.67	N/A	20 h	^[^ ^272^ ^]^
WSe_2_/Zn_0.1_Cd_0.9_S	1T	N/A	2	N/A	10% lactic acid	147.32	39.5%	24 h	^[^ ^273^ ^]^

### 4.2 MPTs Catalysts

Recently, the interests in 2D materials used as catalysts have expanded beyond graphene to include other layered vdW materials. Inspired by the enhancement effect of transition metal phosphides or phosphorsulfides,^[^ ^248^ ^]^ MPTs, or named with metal thio/selenophosphates, including MPS_3_ (M = Fe, Mn, Ni, Cd, Zn) and MPSe_3_ (M = Fe, Mn) are attracting attention this decade. The synergistic P atoms in the chalcogen structure reduce bandgap and increase the conductivity. The surface‐functionalized group, which are [P_2_Ch_6_]^4−^ sites formed by P and chalcogen atoms, has also been reported to assist the H adsorption in HER, thus benefiting the HER activity.^[^ ^248^ ^]^ In the case of free energy, a smaller Δ*G*_H_ with MPTs was obtained compared to the metal sulfide, phosphide, or selenide, indicating their potential in catalytic applications.^[^ ^249^ ^] ,[^ ^250^ ^]^

With size reduced into nanoscale in a 2D structure, MPTs performances better as electrochemical catalysts compared to the bulk form with tuned valance and conductive band position, bandgap, bandgap position, and large surface area. Moreover, 2D MPT material's basal plane remains inert, while uncoordinated sites are active, similar to semiconducting TMD. Lower dimension improves the catalytic property by a higher conductivity and large surface area. Meanwhile, the activity of basal plane has been reported to get promoted by various methods, including vacancies, heteroatoms doping,^[^ ^251^ ^]^ electron and hole doping,^[^ ^162^ ^]^ strain,^[^ ^252^ ^]^ etc.

**Figure 14:**
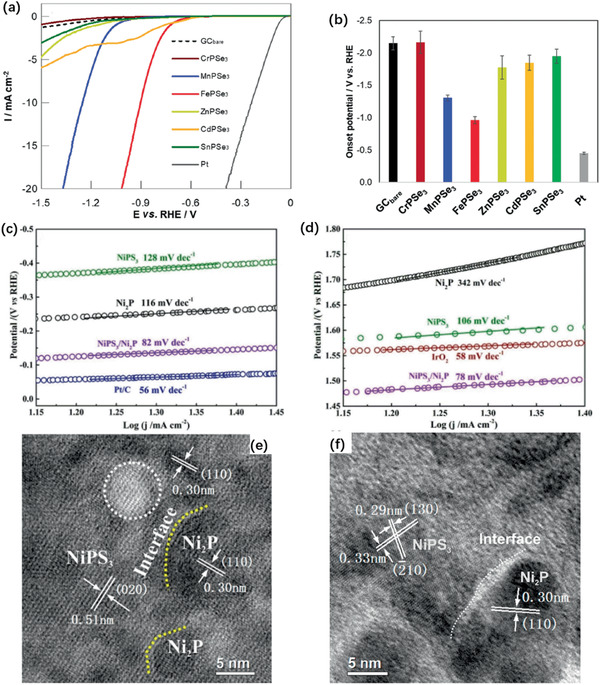
a) SV of HER in 1.0 m KOH and d) the average overpotential at a current density of −10 mA cm^−2^. a,b) Reproduced with permission.^[^ ^157^ ^]^ Copyright 2017, ACS. c,d) Tafel plots for NiPS_3_, Ni_2_P, NiPS_3_/Ni_2_P, PT/C as electrocatalysts for c) HER and d) OER. e,f) TEM images of the NiPS_3_/Ni_2_P heterostructures e) before and f) after long‐term HER testing. c–f) Reproduced with permission.^[^ ^255^ ^]^ Copyright 2019, ACS.

A vital restriction parameter for H_2_ evolution process is the catalyst stability due to the irreversible oxidization of MPT on the surface. It was reported that MPSe_3_ exhibit lower onset potential than MPS_3_ In case of HER. Nonetheless, they yield values that are far from competitive and suffer from inferior stability.^[^ ^54^ ^]^ Meanwhile, MPT suffers the phosphorous/sulfide corrosion, especially for an alkaline solution, bringing FePSe_3_ and MnPSe_3_ up to the front by their outstanding stability. BiPS_4_ lost part of P after used in HER for 100 cycles.^[^ ^54^ ^]^ First, Gusmao et al. report applying MPSe_3_ in HER in alkaline solution, with a low onset potential. Paramagnetic FePSe_3_ and MnPSe_3_ prove to have better performance and stability among other MPSe_3_ in HER with an overpotential of −0.91 mV versus RHE (**Figure** **14**a,b).^[^ ^157^ ^]^

OER is the anodic half‐reaction of water electrolysis and brings other challenges, as discussed in Section 4.1. Active intermediate heterogeneous species, including *OH, *O, and *OOH, have generated and degrade rapidly on MPT nanostructures surface during the reaction.^[^ ^253^ ^]^ Thus activation and maintain active species are in the focus of researchers to achieve stable OER catalyst. Chang et al.^[^ ^254^ ^]^ reported Fe_2_P_2_S_6_ nanostructures with a smaller overpotential (288 mV at 10 mA cm^−2^) in OER compared with FeP (309 mV) and FeS2 (395 mV). The Tafel slopes were calculated to be 45.7, 65.1, and 58.1 mV dec^−1^ respectively, indicating the facilitated OER efficiency. XRD, XPS, and Raman investigation further confirm the oxidized metal species (say FeOOH) as the active catalytic sites for OER.

Likewise, exfoliated Ni‐based MPT is also widely used for its low onset potential. A recent study applied NiPS_3_ heterojunction catalyst used in water‐splitting revealed that it is the epitaxial interface rather than the additive amount has a decisive promotion of the catalytic activity.^[^ ^255^ ^]^ As shown in Figure 14c,d, the onset potential of heterogeneous NiPS_3_/Ni_2_P catalyst is 20 mV lower than NiPS_3_ and Ni_2_P, so as the overpotential and Tafel slope. DFT calculations reveal that the heterojunction accelerates electron transfer due to the built‐in electric field at the epitaxial interfaces thus significantly decreases the kinetic barrier for hydrogen adsorption. Enhanced catalytic efficiency was also seen for OER with lower overpotential (102 mV at a current density of 10 mA cm^−2^) and smaller Tafel slope (78 mV dec^−1^), as shown in Figure 14c,d. Meanwhile, experimental and DFT calculation confirmed that the lower energy barrier and enhanced electric filed at epitaxial interfaces to maximize the electrocatalytic activity of 2D MPTs (Figure 14e,f). Also, the stability of the metallic state of MPT remains challenging.

Higher catalytic efficiency is required for ORR because H_2_ and O_2_ quickly decompose, especially under alkaline conditions. HER attracts researchers' interests to produce a sustainable source of H_2_, while ORR converts chemical energy into electrical energy.^[^ ^256^ ^]^ H_2_O_2_, an essential chemical in industries, can potentially be derived from ORR as well. Generally, the ORR involves either four‐proton‐electron transfers to reduce oxygen to water, desirable for fuel cells for energy conversion, or a two‐proton‐electron pathway, attractive for the production of H_2_ and O_2_.^[^ ^256^ ^]^ From a series of synthesized bulk MPT crystals, MnPS_3_ has a peak attributed to ORR close to Pt (0.28 mV) at a lower cost. Hao et al. reported small Tafel slope value of 62.55 mV dec^−1^ with the few‐layered FePSe_3_ nanosheets catalyst, implying its superior ORR kinetics.^[^ ^257^ ^]^ Different ORR activity of bulk and 2D MPT suggests a negligible effect of low dimension, as reported for TMD. In contrast, MPT catalyst used in the ORR activity is yet to explore. A summarization of MPT used as electro‐catalysts is shown in **Table** **4**.

**Table 4:** Summary of electrochemical catalytic performance of some layered MPT catalysts

	Catalyst	Low‐temperature phase	Thickness [nm]	Electrolyte	Loading [mg cm^−2^]	Overpotential [mV]	Exchange current density [mA cm^−2^]	Tafel slope [mV dec^−1^]	Stability	Ref.
**HER**	Mn_0.05_Ni_0.95_PS_3_	Monoclinic	7.6	1.0 м KOH	1.25	*η*_10_ = −320	166	135	1000 cycles	^[^ ^281^ ^]^
	Ni_0.95_Co_0.05_PS_3_	Monoclinic	6.7	1.0 м KOH	0.51	Η_‐10_ = 71	N/A	77	7 h	^[^ ^282^ ^]^
	Ni_0.9_Fe_0.1_PS_3_	Monoclinic	4.0	1.0 м KOH	0.46	*η*_10_ = 72	N/A	73	50 h	^[^ ^283^ ^]^
	Ni_0.7_Fe_0.3_PS_3_	Monoclinic	1.3	1.0 м KOH	0.25	*η*_10_ = 282	N/A	36.5	50 h	^[^ ^284^ ^]^
	C/NiPS_3_	Monoclinic	1.5	1.0 м KOH	0.41	*η*_10_ = 53.2	0.7	38.2	15 h	^[^ ^251^ ^]^
	NiPS_3_	Monoclinic	0.7	1.0 м KOH	1.0	*η*_10_ = 300	N/A	95	24 h	^[^ ^169^ ^]^
	FePS_3_	Monoclinic	N/A	1.0 м KOH	0.2	*η*_10_ = 175	N/A	137	55 h	^[^ ^254^ ^]^
	FePS_3_@rGO	Monoclinic	0.8	0.5 м H_2_SO_4_	0.15	*η*_10_ = −95	1±0.2	45∼50	1000 cycles	^[^ ^249^ ^]^
**OER**	FePS_3_	Monoclinic	0.7∼1.7	0.5 м KOH	0.15	*η*_10_ = 430	0.16	115	1000 cycles	^[^ ^250^ ^]^
	FePS_3_/FeOOH	Monoclinic	2.96	1.0 м KOH	N/A	390	N/A	58	12 h	^[^ ^155^ ^]^
	FePS_3_	Monoclinic	N/A	1.0 м KOH	0.2	*η*_10_ = 288	N/A	45.7	55 h	^[^ ^254^ ^]^
	NiPS_3_/Ni_2_P	Monoclinic	28	1.0 м KOH	0.56	*η*_10_ = 102	N/A	78	12 h	^[^ ^255^ ^]^
	Mn_0.85_Ni_0.15_PS_3_	Monoclinic	7.6	1.0 м KOH	1.25	*η*_10_ = 350	N/A	151	1000 cycles	^[^ ^281^ ^]^
	Fe‐doped NiPS_3_	Monoclinic	16	1.0 м KOH	N/A	*η*_30_ = 256	N/A	46	12 h	^[^ ^285^ ^]^
	NiPS_3_@NiOOH	Cubic	0.64	0.1 м KOH	0.38	*η*_10_ = 350	N/A	80	160 h	^[^ ^286^ ^]^
	(NiFe)PS_3_	Cubic	10–20	1.0 м KOH	N/A	*η*_10_ = 223	N/A	41.7	N/A	^[^ ^287^ ^]^
	NiPS_3_	Monoclinic	0.6–1.2	1.0 м KOH	1.00	*η*_10_ = 301	N/A	43	20 h	^[^ ^288^ ^]^
	N‐doped C/CoNiPS_3_	Monoclinic	6	1.0 м KOH	3.0	*η*_30_ = 262	N/A	56	28 h	^[^ ^289^ ^]^

An effective effort to make the most of the surface area and conductivity of the 2D MPT nanosheets is to apply them in photocatalytic reaction. Theoretical calculation confirmed MPT high mobility, which is reduced recombination of excited carriers and used as a predictor for high photocatalytic efficiency.^[^ ^274^ ^]^ The bandgaps of these MPX_3_ nanosheets range from 1.3 to 3.5 eV, suggesting solar energy harvesting in a broader range and corresponded outstanding photocatalytic efficiency.^[^ ^66^ ^]^ Incorporating P into the chalcogen structure creates more bandgaps near the Fermi level and reduces the bandgap.^[^ ^274^ ^]^ For this reason, bandgaps of 2D MPT, e.g. FePS_3_^[^ ^275^ ^]^ (2.18 eV), MnPS_3_ (3.14 eV),^[^ ^276^ ^]^ and MnPSe_3_^[^ ^277^ ^]^ (2.32 eV), are appropriate for water splitting, the activation energy of which is 1.23 eV.

Wide bandgaps and vast surface area of 2D MPT materials endow photoelectronic and photocatalytic activities with wide‐ranged light absorption and interfacial reaction activity. Based on MPT 2D materials, some of the efforts have been made to investigate the potential as photocatalysts these years. **Table** **5** gives examples of 2D MPT materials used as photocatalysts recently.

**Table 5:** Summary of light‐induced water‐splitting performance of some 2D MPT catalysts

Catalyst	Low‐temperature phase	Thickness [nm]	Loading	Bandgap [eV]	sacrificial hole scavenger	Solar light	Hydrogen evolution rate [µmol g^−1 ^h^−1^]	Ref.
NiPS_3_	Monoclinic	≈4.9	N/A	1.6	TEOA	400 W Xe lamp	2.6 × 10^3^	^[^ ^280^ ^]^
Ag_0.5_In_0.5_PS_3_	Monoclinic	≈2.1	N/A	2.1	TEOA	400 W Xe lamp	1.9 × 10^3^	^[^ ^280^ ^]^
FePS_3_	Monoclinic	20.6*2 µm	0.05 mg mL^−1^	1.6	TEA, TEOA, EtOH, MeOH	300 W Xe lamp	402.4	^[^ ^290^ ^]^
FePS_3_	Monoclinic	4–8	N/A	2.18	N/A	N/A	290	^[^ ^291^ ^]^
FePS_3_@FTO	Monoclinic	7	0.6 mg cm^−2^	2.0	0.01m Na_2_SO_4_	300 W Xe lamp	305.6	^[^ ^292^ ^]^
NiPS_3_@carbon fiber	Hexagonal	3.5	1.25–1.88	1.96	Na_2_S/Na_2_SO_3_	300 W Xe lamp	74.67	^[^ ^158^ ^]^
MnPSe_3_	Monoclinic	28	≈20	2.0	Sacrificial agent free	300 W Xe lamp	6.5	^[^ ^159^ ^]^

**Figure 15:**
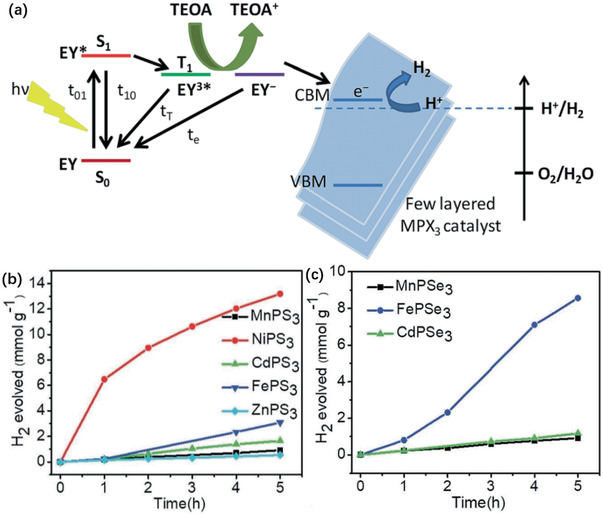
a) Schematic of a plausible mechanism of the H2 evolution reaction activity by MPX3 based photocatalysts. b,c) Hydrogen evolution activities of b) monometallic MPS_3_ and monometallic MPSe_3_ nanosheets. c) Reproduced with permission.^[^ ^280^ ^]^ Copyright 2019, RSC.

However, sulfide is susceptible to photocorrosion (S^2−^ + H → S) and is highly unstable.^[^ ^278^ ^]^ The corrosion can be overcome by rapid depletion or migration of photogenerated holes on the catalyst's valence band.^[^ ^279^ ^]^ As a critical factor, the surface area brings out more activation sites on the surface and better transportation and mobility of reactant. Few layered or even monolayered MPT show a better catalytic efficiency with higher surface area and more active sites. Barua et al. reported an ultrahigh hydrogen evolution rate (2.6 mmol h^−1^ g^−1^) with monolayered NiPS_3_ catalysts in a recent study.^[^ ^280^ ^]^ Other monolayered MPTs, including FePSe_3_, MnPSe_3_, CdPS_3_, etc., also work out as good photocatalyst in HER, as shown in **Figure** **15**a,b.

In summarization, stability like other vdW materials limits the applications of phase transition 2D materials as catalysts. Many of theory 2D crystals are difficult to survive in reality because of the trend of easy corrosion, decomposition, and segregation. The phase transition is also not easy to control that sometimes the transformation happens automatically, making some of the phase challenging to obtain. Meanwhile, 2D phase transition materials are susceptible to contamination, which is highly mobile and hard to notice and detect.

## 5 Summary and Outlook

In this study, the relationship between crystal structure, properties, progress in synthesis, and the catalytic applications of 2D phase transition materials is discussed. The TMD materials usually possess covalent bonding. In contrast, the MPT materials feature comparably strong ionic bonding. From the set of metal elements addressed in this study, Mn, Fe, and Ni have been the most explored, with exciting catalytic water splitting performances. A wide range of methods (e.g., doping and introducing vacancies) have been employed to enhance catalytic efficiencies. Nevertheless, there have been rare examples to clarify the most advantageous preparation method of the mentioned materials given the desired application.

Synthesis routes to the mentioned atomic‐thick layered materials have been developed, while more theory and experimental efforts are required to restrain the reversible phase transition into stable and pure product. For TMDs, CVD is the most promising method, but its difficulty is the need for accurate condition control and optimization (e.g., precursor design, temperature control, atmosphere regulation). Alkali ion intercalation has been extensively studied to induce phase transition of TMDs. This process of inducing phase transition is relatively controllable and partially reversible. However, more theories and experiments are required to gain insights into the mechanism and process of intercalation induced phase transition. Electrostatic gating is promising since it is reversible and nondestructive, whereas the doping concentration and depth are relatively small, which should be further improved. The stress method has a broad prospect, and the stress threshold for phase transition can be regulated by temperature. Thermal treatment is not a good phase transition strategy. It will inevitably introduce defects, and high temperature even damages the structure of materials. Besides, the temperature of the thermal treatment is challenging to control. External irradiation refers to a relatively clean method exhibiting programmable and controllable properties. However, high‐energy particles may cause damage to samples, so external irradiation conditions should be further controlled and optimized. In summary, the preparation strategies of TMDs mainly include the following three challenges: i) Phase transitions are usually reversible. The TMDs of the metallic phase have high conductivity and rich reactivity sites, so they are an excellent electrical catalyst. However, the TMDs of the metallic phase are usually in a metastable structure, so the phase transition from the semiconductor phase to the metallic phase is generally reversible. ii) Some 2D TMDs are unstable due to the influence of oxygen and water in the environment. Therefore, the subsequent stability of the target phase products should be considered in a range of phase transition strategies. iii) Phase purity acts as a vital factor of the catalytic performance of materials.

For MPTs, the preparation methods are mainly CVT method, CVD method, and micromechanical exfoliation method. The CVT method needs to precisely control the reaction conditions, significantly impacting the conversion efficiency and product type. It can be predicted that CVD method is a promising direction. As compared with TMDs, however, MPTs pertain to ternary compounds, the preparation is more difficult to control. Thus, CVD method will face many challenges. Some MPTs have been successfully prepared with CVD method, but compared with TMDs, the controlled growth of MPTs with monolayer, large size, and high uniformity is more difficult to achieve. The main challenge of liquid phase exfoliation in the micromechanical exfoliation method refers to the control of introduced impurities and defects. In addition, few MPTs based on the heavier chalcogen have been reported, which requires creative studies. Finally, the exploration of nonlayered 2D MPTs is another promising direction.

The margin for progress for 2D phase transition material in catalytic applications remains immense. The challenge also remains in fundamental property measurement and application (e.g., predicting/discovering new 2D phase transition material, synthesis control route, poor air stability). The catalyst should further obtain a stabilized phase transition to achieve compatible efficiency with Pt, etc. Though publications on TMD and MPT have surged, this material class remains unclear, particularly when compared with other 2D layered materials. This is principally evident in the current lack of publications on quasi‐2D layers of transition material which hold great promise as catalyst.

## Conflict of Interest

The authors declare no conflict of interest.
